# Chemical profiling, biological evaluation, and QSAR-informed metabolite prioritization of *Hypericum ekerii*: a first report on antioxidant, enzyme inhibitory, and cytotoxic activities

**DOI:** 10.1039/d6ra04698c

**Published:** 2026-09-02

**Authors:** Gokhan Zengin, Sakina Yagi, Mehmet Veysi Cetiz, Abdullahi Ibrahim Uba, Filip Nikolić, Uroš Gašić, Mehmet Maruf Balos, Ahmet Erharman, Nurettin Dogan, Ismail Koyuncu, Ismail Yapıcı, Ilhami Gulcin

**Affiliations:** a Department of Biology, Science Faculty, Selcuk University Konya Türkiye gokhanzengin@selcuk.edu.tr; b Department of Botany, Faculty of Science, University of Khartoum Khartoum Sudan; c Department of Medical Biochemistry, Faculty of Medicine, Harran University Sanliurfa 63290 Türkiye; d Department of Biostatistics and Medical Informatics, Faculty of Medicine, İstinye University 34396 Istanbul Türkiye; e Department of Plant Physiology, Institute for Biological Research “Siniša Stanković”, National Institute of Republic of Serbia, University of Belgrade Bulevar Despota Stefana 142 11108 Belgrade Serbia filip.nikolic@ibiss.bg.ac.rs uros.gasic@ibiss.bg.ac.rs; f Sanlıurfa Provincial Directorate of National Education Sanliurfa Türkiye; g Selcuk University, Faculty of Technology, Department of Computer Engineering Konya Türkiye; h Gümüşhane University Central Research Laboratory Gümüşhane Türkiye; i Department of Chemistry, Science Faculty, Ataturk University Erzurum Türkiye; j Rectorate of Agri İbrahim Çeçen University 04100 Agri Türkiye

## Abstract

*Hypericum ekerii* Yüce and Aytaç, first described in 2017, remains poorly characterized in terms of its phytochemical composition and biological properties. This study investigated aerial-part extracts of *H. ekerii* prepared with ethyl acetate, acetone, 70% acetone–water, and water. The hydroacetone extract showed the highest total phenolic and flavonoid contents. LC/MS analysis enabled the annotation of 99 constituents, including phenolic acids, flavonoids, xanthones, anthraquinones, and acylphloroglucinols. The hydroacetone extract exhibited the strongest radical-scavenging and reducing activities, whereas acetone and ethyl acetate extracts showed the highest metal-chelating capacity. In enzyme assays, hydroacetone was the most active extract against acetylcholinesterase, tyrosinase, and hCA II, while ethyl acetate showed greater inhibition of butyrylcholinesterase and hCA I. The aqueous extract was the most active against α-glucosidase, and hydroacetone and ethyl acetate showed comparable α-amylase inhibition. Cytotoxicity remained limited overall; the lowest IC_50_ value was observed for the ethyl acetate extract against HCT-116 cells (95.09 µg mL^−1^), with weak selectivity (SI = 1.2). A QSAR-informed prioritization framework identified myricetin as the leading cross-layer candidate, *p*-coumaric acid as a target-specific priority metabolite, and ellagic acid, quercetin, and caffeic acid as antioxidant-priority candidates. Molecular docking supported favorable binding profiles for selected metabolites, while molecular dynamics simulations were used to assess the stability of representative complexes. Overall, *H. ekerii* appears to be a chemically diverse species with notable antioxidant and enzyme-inhibitory potential, providing a focused basis for future compound-level validation.

## Abstract 2

These results may provide a scientific basis for developing functional applications that make use of *Hypericum ekerii*. Image produced in part using Google Gemini.

## Graphical abstract

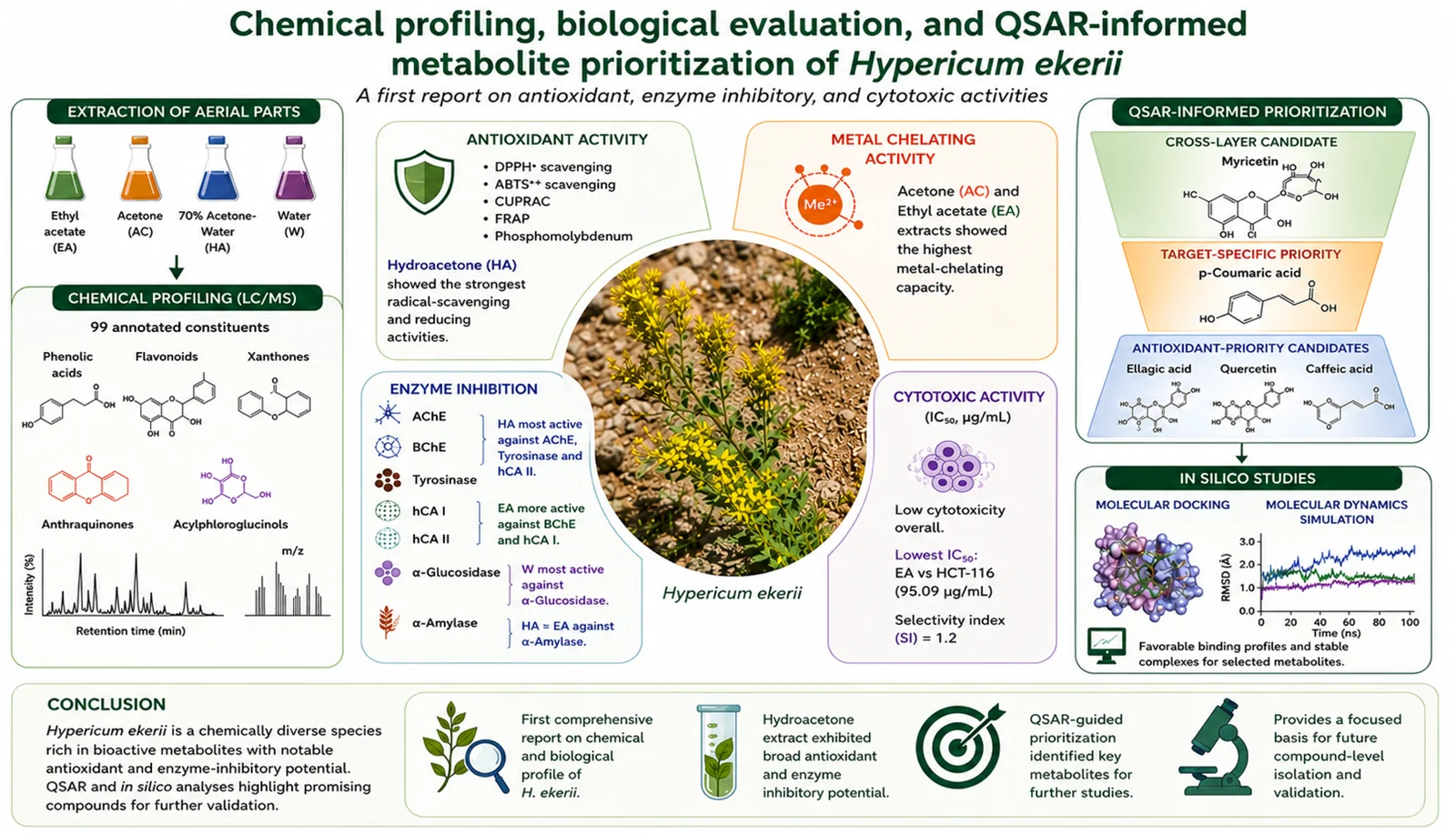

## Introduction

Plants produce a wide range of secondary metabolites with varied biological properties. Many investigations have highlighted the role of plant-derived extracts and compounds in the prevention and management of neurodegenerative disorders, diabetes, infectious diseases, cancer, and other conditions. In addition, plants are considered among the most abundant sources of antioxidants and remain an important reservoir for discovering new bioactive substances.^1^

The genus *Hypericum* L. (family Hypericaceae) includes approximately 500 species, classified into 36 taxonomic sections based on morphological and molecular features.^2^ These species are distributed globally, particularly in temperate and mountainous regions. *Hypericum* species have long been used in traditional medicine systems to treat conditions such as mild depression, wounds, burns, diarrhea, pain, fever, and envenomation from animal bites.^3,4^ Among them, *H. perforatum* (St. John's Wort) is the most well-known species. It is included in European, Chinese, and Indian pharmacopoeias and is widely utilized for alleviating mild depressive symptoms.^3^ Its major bioactive constituents, hypericin and hyperforin, are responsible for its therapeutic effects.^5–7^ Overall, more than 400 natural compounds, primarily phloroglucinols, acylphloroglucinols, xanthones, and benzophenones, have been identified within this genus.^3^*Hypericum* species also exhibit a broad range of biological activities, including cytotoxic, antimicrobial, anti-inflammatory, antioxidant, antifungal, astringent, antihyperglycemic, hepatoprotective, and acetylcholinesterase inhibitory effects.^7–9^

In Turkey, the genus *Hypericum* is represented by 96 species, along with 2 subspecies and 6 varieties, classified into 20 sections. Eastern Turkey and Transcaucasia are regarded as important centers of diversity for this genus.^10^*Hypericum ekerii* Yüce & Aytaç, described as a new species in 2017, is endemic to Tunceli Province and shows close morphological similarity to *H. capitatum*.^11^ Although numerous studies have confirmed the presence of bioactive constituents such as hypericins, flavonoids, and phenolic acids in *Hypericum* species, accounting for their wide range of pharmacological properties, information remains limited for many members of the genus.^7,12,13^ Furthermore, there are currently no reports addressing the chemical composition or biological activities of *H. ekerii*. Although LC/MS-based profiling provides valuable information on the chemical composition of plant extracts, extract-level bioactivity results do not directly indicate which individual metabolites are most likely to contribute to the observed biological effects. This limitation is particularly important in studies involving multiple extracts and numerous tentatively identified compounds, where simple compound listing or abundance-based interpretation may not be sufficient. Therefore, computational prioritization strategies that integrate metabolite distribution, bioactivity patterns, target-specific external QSAR evidence, and applicability-domain assessment may provide additional support for selecting candidate compounds for future validation. Therefore, the present study aims to evaluate the phytochemical profile, antioxidant capacity, enzyme inhibitory and cytotoxic activities of various extracts obtained from the aerial parts of *H. ekerii*. Antioxidant potential was assessed through radical scavenging ability, metal chelation, and reducing power assays. Enzyme inhibition was tested against acetylcholinesterase, butyrylcholinesterase, tyrosinase, α-amylase, α-glucosidase, and human carbonic anhydrase isoenzymes I and II. Additionally, cytotoxic effects were examined using HeLa, A549, and HCT-116 cancer cell lines, along with normal CRL-1459 cells. In addition, a QSAR-informed explainable metabolite prioritization workflow was applied to link the LC/MS-derived metabolite profile with extract-level bioactivity patterns and to tentatively annotate compounds candidate metabolites for future validation. Molecular docking and molecular dynamics simulations were also employed to examine the interaction potential and stability of selected metabolites within the experimentally relevant enzyme targets.

## Materials and methods

### Plant collection

Plant specimens were collected in 2024 from the Duzgun Babadagi Mount (Tunceli, Turkey). Dr Ugur Cakilcioglu carried out the formal botanical classification of the material. A voucher specimen, under code MB-24-62, was deposited at Tunceli University. After collection, the aerial parts were immediately separated and air-dried at room temperature in a shaded, well-ventilated area. After complete drying, the plant material was milled into a fine powder. To ensure chemical integrity and prevent deterioration, the powdered samples were sealed in opaque containers and stored under stable conditions.

### Plant extract preparation

Four solvent systems were used to obtain bioactive constituents: ethyl acetate, acetone, 70% (v/v) acetone-water, and distilled water. For each extraction, 10 g of the prepared plant material was mixed with 200 mL of the corresponding solvent. Organic solvent extractions were performed by maceration for 24 h at 25 ± 2 °C under gentle agitation and protected from direct light. The aqueous extraction was carried out by infusion with distilled water at 95 ± 2 °C for 15 min. Furthermore, to minimize the degradation of photosensitive constituents, particularly the naphthodianthrones (*e.g.*, hypericin derivatives) and phloroglucinols (*e.g.*, hyperforin derivatives) known to occur in *Hypericum* species, all extraction procedures were performed with minimal exposure to direct light. Organic solvent extracts were concentrated under reduced pressure at 40 °C (approximately 150–200 mbar) using a rotary evaporator, whereas the aqueous extract was freeze-dried. The dried extracts were stored in amber glass vials at −20 °C in the dark until analysis (storage period <1 month). Prior to phytochemical and biological assays, dried organic extracts were reconstituted in their respective extraction solvents, while the aqueous extract was dissolved in distilled water to the required concentrations. Fresh solutions were prepared immediately before use.

### Total phenolic and flavonoid contents

Quantification of total phenolics^14^ and total flavonoids^15^ in the extracts was achieved using validated colorimetric techniques, in accordance with established protocols.

In the total phenolic content assay, diluted Folin-Ciocalteu reagent (100 µL, 1 : 9, v/v) was combined with sample solution (50 µL) and vigorously shaken. Na_2_CO_3_ solution (750 µL, 1%) was added after 3 minutes, and the sample absorbance was measured at 760 nm after two hours of room temperature incubation. Milligrams of gallic acid equivalents (mg GAE per g extract) were used to express the total phenolic content (Calibration equation: absorbance = 0.2163 (µg), *R*^2^: 0.9985, 0–5 µg).

The AlCl_3_ technique was used to calculate the total flavonoid content. In short, the same volume of aluminum trichloride (2%) in methanol was combined with the sample solution (100 µL). In a similar manner, 200 µL of sample solution was added to 1 mL of methanol without AlCl_3_ to create a blank. Following a 10 minute incubation period at room temperature, the sample and blank absorbances were measured at 415 nm. The sample's absorbance was deducted from the blank's. The total flavonoid content was measured in milligrams of rutin equivalents (mg RE per g extract) using rutin as a reference standard (Calibration equation: absorbance = 0.13001 (µg) + 0.0506, *R*^2^: 0.9942, 0–20 µg).

### LC/MS experimental part

The chemical analysis of *Hypericum* extracts was conducted using LC/MS (Thermo Scientific™ Vanquish™ Core HPLC system) coupled to the Orbitrap Exploris 120 mass spectrometer (San Jose, CA, USA). All LC/MS parameters are already explained in detail in Stojković *et al*.^16^

LC system was equipped with an Hypersil GOLD™ C18 analytical column (50 × 2.1 mm, 1.9 µm particle size). The injection volume was 5 µL and the flow rate was constant at 300 µL min^−1^. The compounds of interest were eluted with ultrapure water + 0.1% formic acid (A) and acetonitrile (MS grade) + 0.1% formic acid (B): 5% B in the first min; 5–95% B from 1 to 10 min; 95% B from 10 to 12 min; 5% B until 15 min.

The Orbitrap Exploris 120 mass spectrometer was equipped with an HESI source operating in negative ionization mode using the following optimized source parameters: spray voltage 2300 V, sheath gas flow rate 40 arbitrary units (AU); auxiliary gas flow rate 10 AU; sweep gas flow rate 1 AU; vaporizer temperature, 350 °C, and ion transfer tube temperature 325 °C. Full scan MS were monitored from 100 to 1500 *m*/*z* with Orbitrap resolution set to 60 000 FWHM, while data-dependent MS^2^ experiments were conducted at an Orbitrap resolution of 15 000 FWHM with normalized collision energy of CID set to 35%. The dynamic exclusion time was set to 10 s with exclusion from a specific scan after 2 occurrences and intensity threshold was set to 1 × 10^5^. Compound annotation was accepted based on a matching score combining accurate mass (±3 ppm), isotopic pattern fit, and MS/dd-MS^2^ spectral matching against reference online libraries or authentic standards, where available.

Procedural blanks and solvent blanks for each of the four-extraction media were prepared and analyzed alongside the sample batch to monitor potential background interference, solvent impurities, or instrumental memory effects. Solvent blanks were injected immediately after the extract showing the highest signal intensity to assess and confirm negligible carry-over between injections.

The obtained MS data were processed and analyzed using R Studio software (version 2023.09.1, build 494) using enviPick and xcms R packages.^17^ Identification of bioactive compounds was conducted based on their chromatographic behavior and HRMS/MS^2^ data, with comparisons made to standard compounds, when available, and literature data about *Hypericum* species, for tentative identification.^18–21^ The literature review was performed by searching the SciFinder database^22^ using suggested molecular formulas and keywords. Data acquisition was carried out using Xcalibur® data system (Thermo Finnigan, San Jose, CA, USA). To differentiate between samples, hierarchical cluster analysis (HCA) plots were constructed in Morpheus software,^23^ based on the Pearson method of cluster agglomeration, adopting the average linkage method. ChemDraw Ultra (version 12.0, CambridgeSoft., Cambridge, MA, USA) was used as a reference software for drawing the structures of chemical compounds.

### Antioxidant assays

Antioxidant activity was determined using a battery of *in vitro* tests, following the method described by Grochowski *et al.*.^24^ Reducing power was assessed using FRAP and CUPRAC, whereas radical scavenging activity was measured using DPPH and ABTS. Results from these four assays were converted to Trolox equivalents and expressed as mg TE per g of dried extract. The antioxidant capacity was additionally quantified using the phosphomolybdenum (PBD) assay (mmol TE per g), and mg EDTA equivalents per gram (mg EDTAE per g) were used to evaluate metal chelating ability (MCA). All experimental details are given in the SI.

### Enzyme inhibitory activity assays

Using established colorimetric protocols,^24^ the extracts were screened for enzyme inhibition against five relevant targets: acetylcholinesterase (AChE), butyrylcholinesterase (BChE), tyrosinase, α-amylase, and α-glucosidase. To enable comparability across samples, inhibition values were quantified relative to reference standards. Inhibition data were normalized using reference standards, with cholinesterase inhibition expressed as mg GALAE per g, α-amylase and α-glucosidase inhibition as mmol ACAE per g, and tyrosinase inhibition as mg KAE per g. The extracts were also screened for inhibitory activity against human carbonic anhydrase isoenzymes I and II (hCA I and hCA II).^25^ All experimental details are given in the supplemental materials.

### Cytotoxic effect

#### Cell culture

Human HeLa (ATCC CCL-2), A549 (ATCC CCL-185), HCT 116 (ATCC CCL-247), and non-malignant CCD-18Co colon fibroblasts (ATCC CRL-1459) were obtained from the American Type Culture Collection. In accordance with the supplier's recommendations, HeLa, HCT and CCD-18Co cells were maintained in DMEM medium. A549 cells in RPMI-1640 medium. Each complete medium contained 10% fetal bovine serum, 100 IU mL^−1^ penicillin, and 100 µg mL^−1^ streptomycin. Cells were maintained at 37 °C in a humidified atmosphere containing 5% CO_2_. After recovery from a master cell bank, cultures were allowed two recovery passages and experiments were restricted to passages 3–15 after thawing; CCD-18Co cultures were additionally used before population-doubling level 35 because this is a finite-lifespan cell strain. Cultures were screened for mycoplasma by a PCR-based assay after thawing, monthly during routine culture, and within 7 days before each independent experiment; only mycoplasma-negative cultures were used.

Dried EtOAc, acetone, and 70% acetone-water extracts were dissolved at 100 mg mL^−1^ in cell-culture-grade dimethyl sulfoxide (DMSO), whereas the aqueous extract was dissolved at 100 mg mL^−1^ in sterile cell-culture-grade water. Complete dissolution was confirmed before dilution in the appropriate complete culture medium. The maximal final vehicle concentration was 0.2% (v/v), and the vehicle concentration was adjusted to the same level in all concentration groups and matched vehicle controls. Untreated controls, matched vehicle controls, and cell-free extract blanks at every concentration were included on each plate. HeLa, A549, HCT 116, and CCD-18Co cells were seeded at 1 × 10^4^ cells/well in 96-well plates and allowed to attach for 24 h. Cells were then exposed to 0, 2.5, 5, 10, 25, 50, 100, or 200 µg mL^−1^ extract for 24 h. MTT was added to a final concentration of 0.5 mg mL^−1^ and incubated for 4 h. After removal of the medium, formazan crystals were dissolved in 100 µL DMSO, and absorbance was measured at 570 nm with a 690 nm reference wavelength using a Thermo Multiskan GO microplate reader. Cell-free extract blank values were subtracted, and viability was normalized to the matched vehicle control.

Concentration-response data were analyzed by four-parameter logistic nonlinear regression. A numerical IC50 was reported only when the observed concentration range encompassed the 50% viability response; when 50% inhibition was not reached at 200 µg mL^−1^, the result was reported as IC50 >200 µg mL^−1^, without extrapolation. Selectivity index (SI) was calculated as IC50 in CCD-18Co cells divided by IC50 in the corresponding cancer cell line. SI was designated not determinable (ND) when either required IC50 was not measurable within the tested range.

#### Cell-death morphology

To confirm apoptosis morphologically, HCT-116 cells treated with the ethyl acetate extract at a concentration of 100 µg mL^−1^ were selected for fluorescence imaging. Following the incubation period, the treated cells were gently washed with phosphate-buffered saline (PBS) to remove any residual extract and then fixed using 70% ethanol to preserve their structural integrity. After fixation, the cells were rinsed with distilled water to remove traces of ethanol and subsequently stained with a working solution of acridine orange/ethidium bromide (Cat. no.: A6014-E1510, Sigma-Aldrich, Germany)—a dual-fluorescent dye combination widely used to distinguish between viable, apoptotic, and necrotic cells based on their differential uptake of each dye. Fluorescence microscopy was then performed to capture images and visually assess the morphological hallmarks of apoptosis in the treated cell population.

#### Cell-death profiling

To further investigate the apoptotic effects of the extracts, we utilized the commercially available FITC Annexin V Apoptosis Detection Kit I (BD Biosciences, New Jersey, USA), following the manufacturer's recommended protocol. HCT-116 cells were seeded into 6-well plates at a density of 5 × 10^5^ cells per well and allowed to adhere overnight. The cells were then treated with the ethyl acetate extract at a concentration of 100 µg mL^−1^ and incubated for a further 24 hours.

Following treatment, the cells were detached using trypsin and carefully transferred into new tubes at a concentration of 1 × 10^6^ cells per mL in 1× binding buffer. Each tube was then gently mixed and incubated at room temperature for 15 minutes in the dark. Subsequently, 5 µL of FITC-conjugated Annexin V and 5 µL of Propidium Iodide (PI) were added to each tube to label apoptotic and necrotic cell populations, respectively. To finalize the staining protocol, 100 µL of 1× binding buffer was added to each sample, followed by centrifugation at 1200 rpm for 5 minutes to pellet the cells. The stained cell populations were then immediately analyzed using a flow cytometer (BD, New Jersey, USA) to quantitatively distinguish between viable, early apoptotic, late apoptotic, and necrotic cells.

#### Computational prioritization of annotated metabolites

Annotated metabolites were prioritized using ADEFRank, an applicability domain-aware workflow integrating extract-level bioactivity patterns with external QSAR predictions. Since the *H. ekerii* dataset included only four extracts, QSAR models were not trained on the extract data. Instead, target-specific ChEMBL datasets,^26^ were curated for carbonic anhydrase isoforms, acetylcholinesterase, butyrylcholinesterase, and mushroom tyrosinase. Structures were standardized with RDKit,^27^ activities were converted to pActivity, and compounds with pActivity ≥ 6.0 were labeled active. Morgan fingerprints^28^ and physicochemical descriptors were used to train Random Forest classifiers,^29^ evaluated by random and Murcko scaffold splits.^30^ The final models scored the annotated metabolites, and prediction reliability was judged by maximum Tanimoto similarity to training compounds. Antioxidant prioritization was assessed separately from single-target QSAR using structural plausibility and extract-level antioxidant concordance. These evidence layers were combined within the ADEFRank framework. In the extract-level pattern component, peak-area values were normalized separately within each metabolite across the four extracts and were used only to represent the relative distribution of the same candidate among the extraction solvents; they were not interpreted as quantitative cross-compound abundance. All predictions and candidate priorities were interpreted only as hypothesis-generating results requiring future compound-level validation.

#### Molecular docking

Molecular docking studies were undertaken to characterize the binding behavior of the major phytoconstituents tentatively annotated in the aerial extracts of *Hypericym ekerii* against a broad spectrum of biologically relevant targets. The selection of protein targets was guided by their direct association with experimentally observed enzyme-inhibitory effects of the extracts. Human AChE, BChE, α-amylase, α-glucosidase, tyrosinase, hCAI, and hCAII were chosen because dysregulation of these enzymes is closely linked to disorders such as Alzheimer's disease, diabetes mellitus, and glaucoma, where enzyme inhibition remains a recognized therapeutic strategy.^31–34^ The metabolites investigated represented diverse phytochemical categories, including flavonoids, phenolic derivatives, and glycosylated compounds. Three-dimensional ligand structures were retrieved from established public databases, primarily PubChem (https://pubchem.ncbi.nlm.nih.gov/) and SpectraBase (https://spectrabase.com/), prior to computational analysis. Detailed information on protein structures, Protein Data Bank (PDB) IDs, and docking parameters is summarized in Table S1.

All protein structures were refined using BIOVIA Discovery Studio and AutoDockTools (v4.2.6) before docking. The best receptor preparation was achieved by removal of the crystallographic water molecules, addition of polar hydrogens and assignment of Gasteiger charges. The ligand geometries were then energy-minimized in Avogadro (v1.2.0) to obtain stable conformations suitable for docking calculations. Docking calculations were performed using AutoDock Vina (v1.1.2) with an exhaustiveness value of 32 to improve the reliability of conformational sampling and pose prediction.^35–37^ Putative active-site cavities were identified using the crystallized ligand in each crystal complex (Table S1). The docking workflow was validated by redocking native co-crystallized ligands into their original binding sites. The resulting docked conformations were compared with experimentally resolved poses by calculating root meaning square deviation (RMSD) values.^38,39^ Following docking, interaction profiles were analyzed using the Protein–Ligand Interaction Profiler (PLIP), allowing detailed characterization of hydrogen bonding, hydrophobic contacts, and aromatic or π-mediated interactions.^14,15^

#### Molecular dynamics simulation

After docking analyses, MD simulations were run to investigate the temporal stability and conformational behavior of the selected protein–ligand complexes. Initial system preparation was carried out using the CHARMM-GUI platform.^40^ Proteins and ligands were parametrized by the CHARMM36m force field, and explicit solvation was performed with the TIP3P water model. In each simulation system, counterions and 0.15 M NaCl were added to reproduce physiological ionic conditions and to maintain charge neutrality.

Short-range nonbonded interactions were treated using the Verlet cutoff scheme. Constraints on covalent bonds containing hydrogen atoms were enforced by the LINCS algorithm. Long-range electrostatic interactions were treated using the Particle Mesh Ewald (PME) method. Each system was energy-minimized prior to equilibration using the steepest descent algorithm until the maximum force fell below 1000 kJ mol^−1^ nm^−1^, thereby removing steric clashes and unfavorable contacts.^41,42^

Subsequently, the system was equilibrated in two steps: first at constant particle number, volume and temperature (NVT) and then at constant particle number, pressure and temperature (NPT) at 310 K. Then production simulations were performed for 100 ns using GROMACS (version 2023).^43,44^ The resulting trajectories were analyzed to assess structural stability, residue flexibility, solvent accessibility, hydrogen-bond persistence, and ligand interaction dynamics throughout the simulation. This integrated computational strategy enabled a robust assessment of ligand-binding affinity and interaction stability across the selected targets.

#### Statistical analysis

Triplicate measurements were taken for all experiments, and differences across extract samples were tested for statistical significance. Analysis was performed using one-way ANOVA (GraphPad Prism, version 9.2), followed by Tukey's post hoc test to compare individual groups. A *p*-value of less than 0.05 was considered statistically significant. Concentration–response curves for human carbonic anhydrase isoenzymes I and II inhibitions were analyzed by nonlinear regression using the [inhibitor] *versus* normalized response model with a variable Hill slope (four-parameter logistic model), and IC_50_ values were calculated from the fitted curves. Hill slope values and 95% confidence intervals were obtained from the nonlinear regression analysis.

## Results and discussion

### Total phenolic (TPC) and flavonoids (TFC) contents

The TPC of the various extracts obtained from the aerial parts of *Hypericum ekerii* ranged from 44.87 to 109.70 mg GAE per g, while the TFC varied between 17.29 and 52.31 mg RE per g (Table 1). Among the extracts, the hydroacetone extract showed the highest levels of both TPC and TFC, whereas the EtOAc extract exhibited the lowest values. Moreover, the hydroacetone extract yielded TPC and TFC values that were approximately 1.8 and 1.4 times higher, respectively, than those obtained from the acetone extract. Previous research indicates that hydroacetone is particularly effective for extracting phenolic compounds. For instance, Mokrani and Madani^45^ reported that, based on total phenolic content (TPC) and antioxidant activity in *Prunus persica* fruit, the optimal extraction condition was 60% acetone. Similarly, El Mannoubi^46^ observed that although an 80% acetone extract of *Opuntia stricta* fruit resulted in the lowest extraction yield, it exhibited the highest TPC, total flavonoid content (TFC), and antioxidant activity.

**Table 1:** Extraction yields (%), total phenolic and flavonoid contents of *Hypericum ekerii* aerial parts extracts^a^

Extracts	Extraction yields (%)	TPC^b^ (mg GAE per g extract)	TFC^c^ (mg RE per g extract)
EA	4.20	44.87 ± 0.46^d^	17.29 ± 0.99^c^
Acetone	5.83	62.25 ± 0.71^c^	38.53 ± 0.48^b^
Acetone/Water	20.79	109.70 ± 1.17^a^	52.31 ± 0.73^a^
Water	15.83	83.93 ± 0.75^b^	38.17 ± 0.19^b^

a Values are means ± SD of three measurements. Means within each column with different letters (a–d) differ significantly (*p* ≤ 0.05).

b mg Gallic acid equivalents per gram of extract.

c mg Rutin equivalents per gram of extract.

### LC/MS result and discussion part

The four *Hypericum* extracts represent four independent extractions performed using different solvents on separate aliquots of the same powdered plant material batch. The genus *Hypericum* is globally recognized for its complex and diverse specialized metabolites, characteristically featuring a co-occurrence of polar polyphenols and lipophilic prenylated derivatives.^47^ Extensive literature data confirms that the pharmacological and chemotaxonomic profile of these plants is primarily governed by five core phytochemical classes: 1) polycyclic quinones or naphthodianthrones (*e.g.* hypericin and pseudohypericin) biosynthetically linked to dimeric anthraquinones like skyrin^48,49^ polycyclic polyprenylated acylphloroglucinols (such as colupulone and adhyperfirin derivatives)^50^ flavonoid *O*- and *C*-glycosides along with their free aglycones and flavan-3-ols^51,52^ specialized oxygenated xanthones (including mangiferin)^53^ various hydroxybenzoic and hydroxycinnamic acids.^54^

Chemical analysis of *Hypericum ekerii* extracts, adopted in our research, tentatively annotated 99 bioactive compounds. The structures of the compounds were proposed based on a review of the literature on *Hypericum* metabolites, where available. The chemical structures of the specified bioactive compounds (hypericin, myricetin-3-*O*-hexuronide, quercetin-3-*O*-hexoside, myricetin, and apigenin) tentatively annotated in *Hypericum* samples are presented in Fig. S1. Table S2 lists all tentatively annotated compounds, divided into several groups according to their basic chemical structures: hydroxybenzoic acids (10 compounds), hydroxycinnamic acids (8 compounds), flavan-3-ols (5 compounds), flavonoid *C*-glycosides (7 compounds), flavonoid *C*-glycosides (26 compounds), flavonoid aglycones (7 compounds), xanthones (6 compounds), anthraquinones (5 compounds), acylphloroglucinols (11 compounds) and the other metabolites (14 compounds). In the same table, there are also references that confirm the previous tentatively annotation of certain compounds in some of *Hypericum* species. Base peak chromatograms of four investigated *Hypericum* samples, obtained with different extraction solvents are depicted in Fig. S2.

To compare the distribution of tentatively annotated compounds across the given extraction solvents, hierarchical cluster analysis (HCA) was performed on peak area values (Table S3) obtained from full-scan MS spectra (Fig. S3). Prior to analysis, peak area values were normalized across the extracts. It is important to note that direct comparisons were strictly limited to evaluating the recovery of the same individual analyte across the four different extraction solvents, rather than cross-comparing absolute abundances among chemically diverse metabolites. Consequently, the relative peak intensities are discussed solely as indicators of extraction efficiency for specific compounds depending on the solvent used, avoiding misleading quantitative rankings between structurally unrelated metabolites. The LC-MS profiling of various *Hypericum ekerii* extracts (acetone/water, acetone, ethyl acetate, and pure water) provided a comprehensive overview of the chemical architecture and distribution of secondary metabolites within this species. The results demonstrate a clear correlation between solvent polarity and extraction efficiency across distinct phytochemical classes (Fig. S3).

Dihydroxybenzoic acid hexoside and free dihydroxybenzoic acid emerged as the quantitatively dominant constituents within this class, showing massive peak intensities in the highly polar extraction media, particularly in the acetone/water and pure water systems. Regarding hydroxycinnamic acids, 5-*O*-caffeoylquinic acid (chlorogenic acid) and 5-*O*-coumaroylquinic acid exhibited exceptionally high abundance in the acetone/water and aqueous extracts, whereas their concentrations dropped drastically in the less polar ethyl acetate medium.

Flavonoid *O*-glycosides (quercetin 3-*O*-hexuronide, quercetin 3-*O*-hexoside, and myricetin 3-*O*-hexoside) dominated the polar profile, reaching their peak abundance in acetone and acetone/water mixtures. Luteolin 8 C-hexoside showed high accumulation in acetone/water and acetone, while remaining nearly undetected in ethyl acetate. The absence of specific C-glycosides (apigenin 6,8-di-*O*-hexoside) in the ethyl acetate fraction underlines their strongly hydrophilic nature. Flavonoid aglycones quercetin, myricetin, kaempferol), unlike their glycosylated counterparts, displayed a more uniform distribution across the organic solvents, with quercetin maximizing in the acetone/water matrix.

Mangiferin, a highly hydrophilic xanthone *C*-glycoside, was selectively extracted only by acetone/water and acetone, disappearing below the detection limit in pure water and ethyl acetate. Conversely, less polar methylated derivatives like trihydroxydimethoxyxanthone and dihydroxydimethoxyxanthone displayed a strong affinity for ethyl acetate and acetone.

From the group of anthraquinones and naphthodianthrones, skyrin (dimeric anthraquinone) stands out showing enormous peak intensities in the acetone and ethyl acetate extracts, while hypericin and pseudohypericin exhibited unique solvent behavior. Hypericin was detected exclusively in the acetone/water mixture and was completely missing from all other solvents. Pseudohypericin, which features an extra hydrophilic hydroxyl group, was successfully recovered in acetone/water, water, and acetone, but remained absent in the lipophilic ethyl acetate fraction.

This class of highly lipophilic prenylated compounds, such as acylphloroglucinols, demonstrated absolute dominance in the ethyl acetate extract. Some compounds, tentatively annotated as colupulone, hypermongone derivatives, tomoeone H, and adhyperfirin reached extreme peak intensities in ethyl acetate, but dropped to negligible levels or total absence in pure water. Due to its low polarity and lack of hydrogen-bonding disruption, ethyl acetate proved to be a superior, protective solvent for extracting unstable acylphloroglucinol derivatives.

The LC-MS profiling confirms that *Hypericum ekerii* possesses a rich and highly specialized metabolomic fingerprint. Solvent selection acts as a strict chemical gatekeeper: if the analytical goal focuses on polar polyphenols, flavonoid glycosides, and naphthodianthrones, an acetone/water system provides the most balanced recovery. However, for the targeted screening or isolation of bioactive acylphloroglucinols and hydrophobic xanthones, ethyl acetate is an indispensable extraction medium.

Based on this LC/MS-derived metabolite profile, the tentatively annotated compounds were used as input to the QSAR-informed explainable metabolite prioritization workflow. This additional analysis was performed to move beyond compound listing and to support the selection of candidate metabolites that may better explain the observed extract-level bioactivity patterns.

### Antioxidant activity

The antioxidant activity of the different extracts obtained from the aerial parts of *H. ekerii* was evaluated using six complementary assays (Table 2). The DPPH and ABTS assays assessed the free radical scavenging capacity of the extracts. The FRAP and CUPRAC assays were used to determine the ion-reducing power, while the phosphomolybdenum assay measured the ability of the extracts to reduce Mo(vi) to Mo(v). The metal chelating activity was evaluated based on the iron chelating capacity of the extracts. The hydroacetone extract exhibited the strongest radical scavenging activity and ion-reducing power in the four assays (DPPH = 184.50 mg TE g; ABTS = 380.78 mg TE g; CUPRAC = 505,54 mg TE g; FRAP = 297.72 mg TE g^−1^), followed by the aqueous extract. Interestingly, despite its weaker performance in radical scavenging and reducing assays, the acetone extract exhibited a high total antioxidant capacity in the phosphomolybdenum assay (2.05 mmol TE g^−1^), comparable to that of the hydroacetone extract (2.08 mmol TE g^−1^), with no statistically significant difference (*p* ≥ 0.05). This suggests that different classes of antioxidant compounds may be contributing to activity depending on the assay mechanism. Furthermore, the acetone and EtOAc extracts exhibited the strongest metal chelating activity (25.99 and 25.46 mg EDTAE/g, respectively; *p* ≥ 0.05), indicating their potential effectiveness in inhibiting metal-catalyzed oxidative processes. This highlights the importance of considering multiple antioxidant mechanisms when evaluating plant extracts, as activity may vary depending on the dominant compounds and their modes of action.

**Table 2:** Antioxidant activity of *Hypericum ekerii* aerial parts extracts^a^

Extracts	DPPH (mg TE per g extract)^b^	ABTS (mg TE per g extract)^b^	CUPRAC (mg TE per g extract)^b^	FRAP (mg TE per g extract)^b^	Metal chelating (mg EDTAE per g extract)^c^	PBD (mmol TE per g extract)^d^
EA	20.68 ± 0.49^c^	52.16 ± 0.72^c^	91.87 ± 1.58^c^	38.87 ± 0.55^c^	25.99 ± 0.41^a^	1.89 ± 0.08^b^
Acetone	9.82 ± 0.31^d^	33.22 ± 1.10^d^	52.98 ± 1.89^d^	26.46 ± 0.30^d^	25.46 ± 0.45^a^	2.05 ± 0.03^a^
Acetone/water	184.50 ± 5.07^a^	380.78 ± 6.20^a^	505.54 ± 2.75^a^	297.72 ± 2.40^a^	9.08 ± 0.35^c^	2.08 ± 0.01^a^
Water	94.99 ± 1.96^b^	198.65 ± 3.61^b^	287.89 ± 6.29^b^	180.42 ± 3.60^b^	13.33 ± 0.55^b^	1.57 ± 0.55^c^

a Values are means ± SD of three measurements. Means within each column with different letters (a–d) differ significantly (*p* ≤ 0.05).

b mg Trolox equivalents per g of extract.

c mg EDTAE per g of extract.

d mmol Trolox equivalents per g of extract.

Previous studies on *Hypericum* species have attributed antioxidant activity primarily to flavonoids and related phenolic compounds. For instance, compounds such as cariphenone A (isolated from *H. carinatum*) and 5,7-dihydroxy-2-isobutyl-4*H*-chromen-4-one (from *H. petiolulatum*) have been reported to possess significant antioxidant properties.^55,56^ Additionally, flavonoids including quercetin, hyperoside, quercetin-3-*O*-β-D-glucoside, kaempferol, and rutin isolated from *H. ascyron* have demonstrated strong DPPH radical scavenging activity.^57,58^ These findings are consistent with the present study, in which a substantial number of flavonoids and phenolic acids, well-known for their antioxidant properties, were tentatively annotated in the extracts. However, the relatively low antioxidant activity of the acetone extract, despite its considerable TPC and TFC, may be explained by the predominance of flavonoids in glycosidic forms. Glycosylation can reduce the accessibility of hydroxyl groups responsible for radical scavenging, thereby diminishing antioxidant efficiency in certain assays.^59^

### Enzyme inhibitory activity

The enzyme inhibitory potential of extracts from the aerial parts of *H. ekerii* was evaluated against a panel of biologically relevant enzymes, including acetylcholinesterase (AChE), butyrylcholinesterase (BChE), tyrosinase (Tyr), α-amylase, α-glucosidase, and human carbonic anhydrase isoenzymes (CA I and II). The results (Tables 3 and 4) revealed that the inhibitory activities varied notably depending on both the enzyme target and the extraction solvent. Among the tested samples, the hydroacetone extract exhibited the strongest inhibitory effect against AChE (2.57 mg GALE per g), while the ethyl acetate (EtOAc) extract showed the highest activity against BChE (3.41 mg GALE per g). The acetone extract also demonstrated considerable inhibition of both AChE (2.46 mg GALE per g) and BChE (3.16 mg GALE per g), suggesting that semi-polar solvents may be particularly effective in extracting cholinesterase-inhibiting constituents. All extracts displayed substantial tyrosinase inhibitory activity, with the hydroacetone extract being the most active (71.49 mg KAE per g), followed by the organic solvent extracts. In contrast, the extracts showed relatively weaker inhibition against carbohydrate-hydrolyzing enzymes. The aqueous extract exhibited the strongest α-glucosidase inhibition (1.28 mmol ACAE per g), followed closely by the hydroacetone extract (1.19 mmol ACAE per g). Regarding α-amylase inhibition, the hydroacetone and EtOAc extracts showed comparable and the highest activities (0.72 and 0.73 mmol ACAE per g, respectively).

**Table 3:** Enzyme inhibitory activity of *Hypericum ekerii* aerial parts extracts^a^

Extracts	AChE^b^ (mg GALAE per g extract)	BChE^b^ (mg GALAE per g extract)	Tyrosinase^c^ (mg KAE per g extract)	α-Amylase^d^ (mmol ACAE per g extract)	α-Glucosidase^d^ (mmol ACAE per g extract)
EA	2.34 ± 0.02^b^	3.41 ± 0.25^a^	51.47 ± 1.45^b^	0.73 ± 0.02^a^	0.35 ± 0.02^c^
Acetone	2.46 ± 0.09 ^ab^	3.16 ± 0.98 ^ab^	55.42 ± 0.62^b^	0.67 ± 0.02^b^	0.38 ± 0.07^c^
Acetone/Water	2.57 ± 0.04^a^	1.41 ± 0.10^c^	71.49 ± 0.16^a^	0.72 ± 0.01^a^	1.19 ± 0.01^b^
Water	2.10 ± 0.04^c^	3.60 ± 0.23^b^	47.96 ± 0.63^c^	0.06 ± 0.01^c^	1.28 ± 0.03^a^

a Values are means ± SD of three measurements. Means within each column with different letters (a–d) differ significantly (*p* ≤ 0.05).

b mg Galantamine equivalents per g of extract.

c mg Kojic acid per g of extract.

d mmol Acarbose equivalents per g of extract.

**Table 4:** Human carbonic anhydrase isoenzymes (hCA) inhibition of *Hypericum ekerii* aerial parts extracts (IC_50_ were expressed as µg mL^−1^). AZA; acetazolamide

Extract name	hCA I		hCA II	
	IC_50_	*r* ^2^	IC_50_	*r* ^2^
EA	48.21 ± 3.81	0.9821	26.93 ± 2.15	0.9932
Acetone	81.62 ± 2.20	0.9925	36.62 ± 1.66	0.9956
Acetone/water	104.00 ± 2.85	0.9876	21.65 ± 4.45	0.9800
Water	147.00 ± 9.45	0.9036	74.17 ± 2.84	0.9900
AZA	82.50 ± 3.75	0.9850	116.00 ± 4.56	0.9683

The extracts exhibited also stronger inhibitory activity against hCA II (IC_50_ = 21.65–74.17 µg mL^−1^) than against hCA I (IC_50_ = 48.21–147.00 µg mL^−1^), indicating a greater selectivity toward the hCA II isoenzyme. Among the tested samples, the hydroacetone extract showed the most potent inhibition of hCA II, followed by the ethyl acetate (EA) and acetone extracts. Notably, their IC_50_ values were lower, respectively, than that of the standard control (AZA), highlighting their superior inhibitory potential.

In addition, the EA extract demonstrated remarkable activity against hCA I, more effective than the standard control. These findings suggest that *H. ekerii* extracts, particularly those obtained with hydroacetone and EA, may serve as promising sources of carbonic anhydrase inhibitors with potential pharmacological relevance. These findings suggest that *H. ekerii* represents a promising natural source of enzyme inhibitors with potential applications in the management of neurodegenerative disorders, hyperpigmentation, and metabolic diseases.

As a structure–activity insight, the enzyme inhibitory effects observed for the tested extracts can likely be attributed to their major constituents. Based on peak areas, quinic acid and quercetin-3-hexuronide were the dominant compounds in the acetone-water extract, and both are plausible contributors to its AChE inhibitory activity: Yılmaz *et al.* reported favorable *in silico* binding between quinic acid and AChE,^60^ while Güven *et al.* identified quercetin-3-hexuronide as the main component of *Alchemilla pseudocartalinica* and showed it binds AChE effectively.^61^ Quinic acid may also underlie the tyrosinase inhibitory activity seen here, through interactions with the enzyme's active site and catalytic copper center.^62^ Quercetin-3-hexuronide showed a similar effect, inhibiting tyrosinase with an IC50 of 86.60 mM, and it has separately been reported to inhibit glucosidase strongly as well.^63^ In the literature, previous studies on *Hypericum* species have highlighted the role of hyperforin derivatives in neuroprotection. For instance, Hyperuralone C and D, isolated from *H. uralum*, have been reported to exhibit significant anti-AChE activity.^64^.Although these specific compounds were not found in the present study, other bioactive constituents, such as kaempferol and quercetin, were detected and are well known for their cholinesterase inhibitory properties (Shi *et al.*, 2023; Liao *et al.*, 2022). In addition, these flavonoids have been reported to possess notable tyrosinase inhibitory activity.^65,66^ Other phenolic compounds, tentatively annotated in the extracts, including caffeic acid and gallic acid, have also been associated with tyrosinase inhibition (^67,68^. Furthermore, flavonoids such as quercetin and kaempferol, along with triterpenoids like ursolic acid (reported in *H. ascyron*), have demonstrated significant glycosidase inhibitory activity.^58^ Similarly, hypemonones A and E isolated from *H. monogynum* were shown to exert moderate inhibitory effects on α-glucosidase.^69^ Considering the current results alongside earlier findings, the observed inhibitory activity is more likely driven by synergistic or additive interactions among multiple phytochemicals than by any single compound acting alone. Thus, future studies combining targeted quantitative metabolomics with bioactivity-guided fractionation will be needed to pinpoint the specific metabolites responsible for each enzyme inhibitory activity observed here.

### Cytotoxic effect

The cytotoxic effects of extracts obtained from the aerial parts of *Hypericum ekerii* were evaluated against cervical adenocarcinoma (HeLa), human lung carcinoma (A549), and colorectal cancer (HCT-116) cell lines, together with non-cancerous human colon cells (CRL-1459) (Table 5).

**Table 5:** Cytotoxic effects of *Hypericum ekerii* aerial parts extracts on cancer and normal cell lines (IC_50_ µg mL^−1^)

Extracts	HELA	A549	HCT-116	CRL-1459
EtOAc	158, 47	119, 21	95, 09	109, 62
Acetone	146, 27	141, 99	105, 18	104, 18
Acetone/water	126, 15	127, 61	124, 87	124, 12
Water	>200	>200	>200	>200

The extracts generally displayed limited cytotoxicity. Among extract-cell line combinations for which the 50% response was reached within the tested range, the lowest IC_50_ was observed for the EtOAc extract against HCT-116 cells (95.09 µg mL^−1^), followed by the acetone extract against the same cell line (105.18 µg mL^−1^). In contrast, the aqueous extract did not reduce viability to 50% in any cell line at the highest tested concentration and was therefore reported as IC_50_ > 200 µg mL^−1^ rather than as an extrapolated point estimate. Calculable SI values ranged from 0.7 to 1.2 (Table 6), indicating no pronounced cancer-cell selectivity. The SI of the aqueous extract was not determined because the required IC_50_ values were not observed within the tested concentration range. Because the EtOAc extract produced the lowest measured IC50 in HCT-116 cells (95.09 µg mL^−1^), this extract-cell combination was selected for exploratory cell-death profiling at 100 µg mL^−1^, a concentration close to the measured IC_50_ and within the tested range. This selection was intended to characterize the phenotype underlying the observed reduction in MTT signal and should not be interpreted as evidence of strong cancer-cell selectivity. AO/EB staining showed reduced viable-cell density and an increase in orange/red-stained damaged cells (Fig. 1). Annexin V/PI analysis showed a decrease in the live-cell population from 96.4% to 35.8%, accompanied by an increase in the membrane-compromised/necrotic fraction from 1.0% to 60.8% (Fig. 2). Early apoptotic cells remained low, and late apoptotic cells increased only slightly. Thus, under these conditions, the response was dominated by PI-positive membrane-compromised cellular injury rather than a marked apoptotic shift (Fig. 3). Taken together, these findings indicate that *H. ekerii* extracts, particularly the EtOAc fraction, can induce detectable cellular damage at the tested concentration, but the overall IC_50_ and selectivity index profiles do not support strong or selective cytotoxic activity. Previous studies have likewise reported variable and generally moderate cytotoxic effects among *Hypericum* species.^70–72^

**Table 6:** Selective index evaluation of cancerous cells compared to CRL-1459 normal cells of *Hypericum ekerii* extracts

Cells	HELA	A549	HCT-116
EtOAc	0, 7	0, 9	1, 2
Acetone	0, 7	0,7	1, 0
Acetone/water	1, 0	1, 0	1, 0
Water	N.D	N.D	N.D

**Fig. 1:**
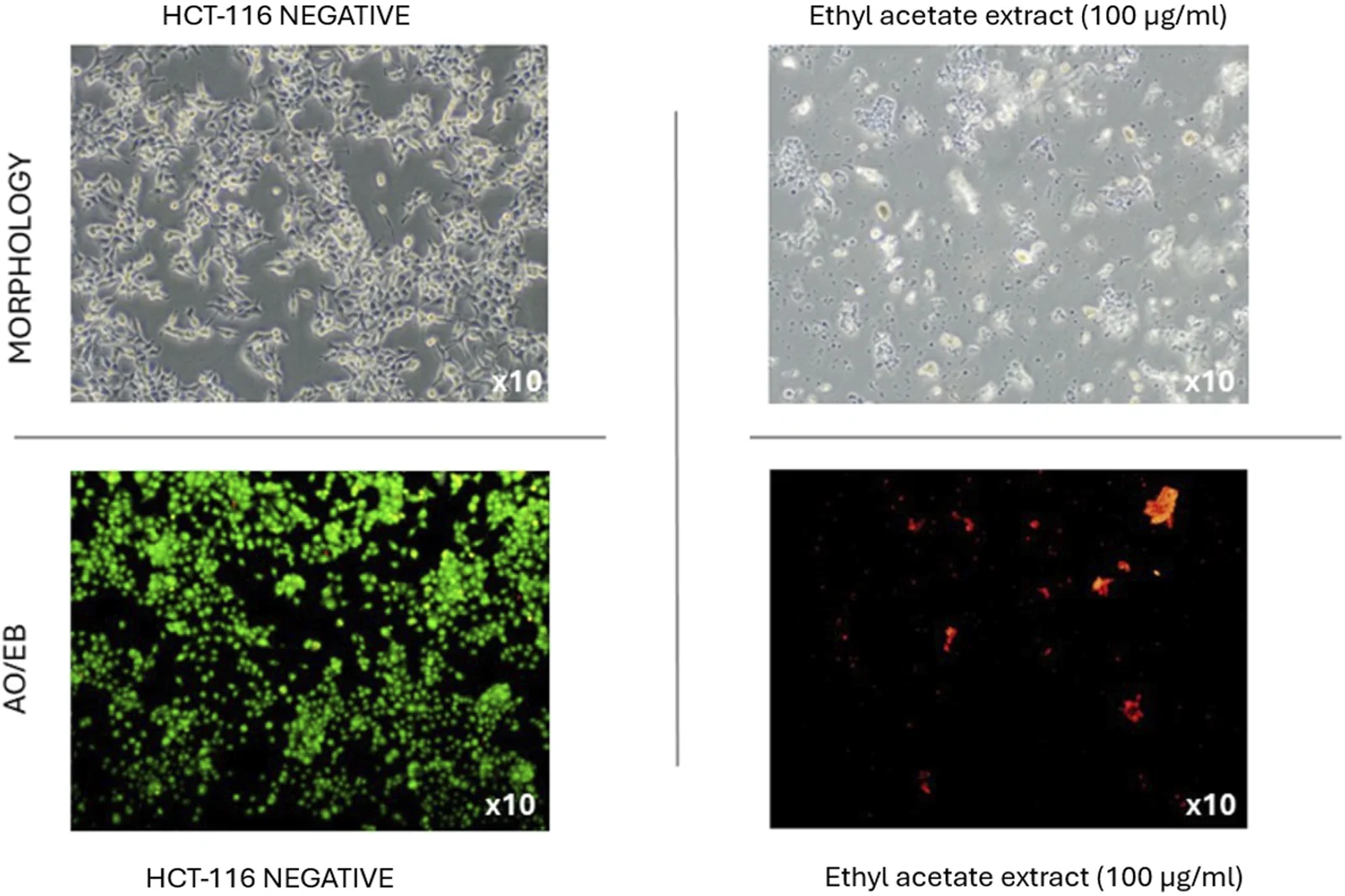
AO/EB staining after ethyl acetate extract (100 µg mL^−1^) applied to HCT-116 cell.

**Fig. 2:**
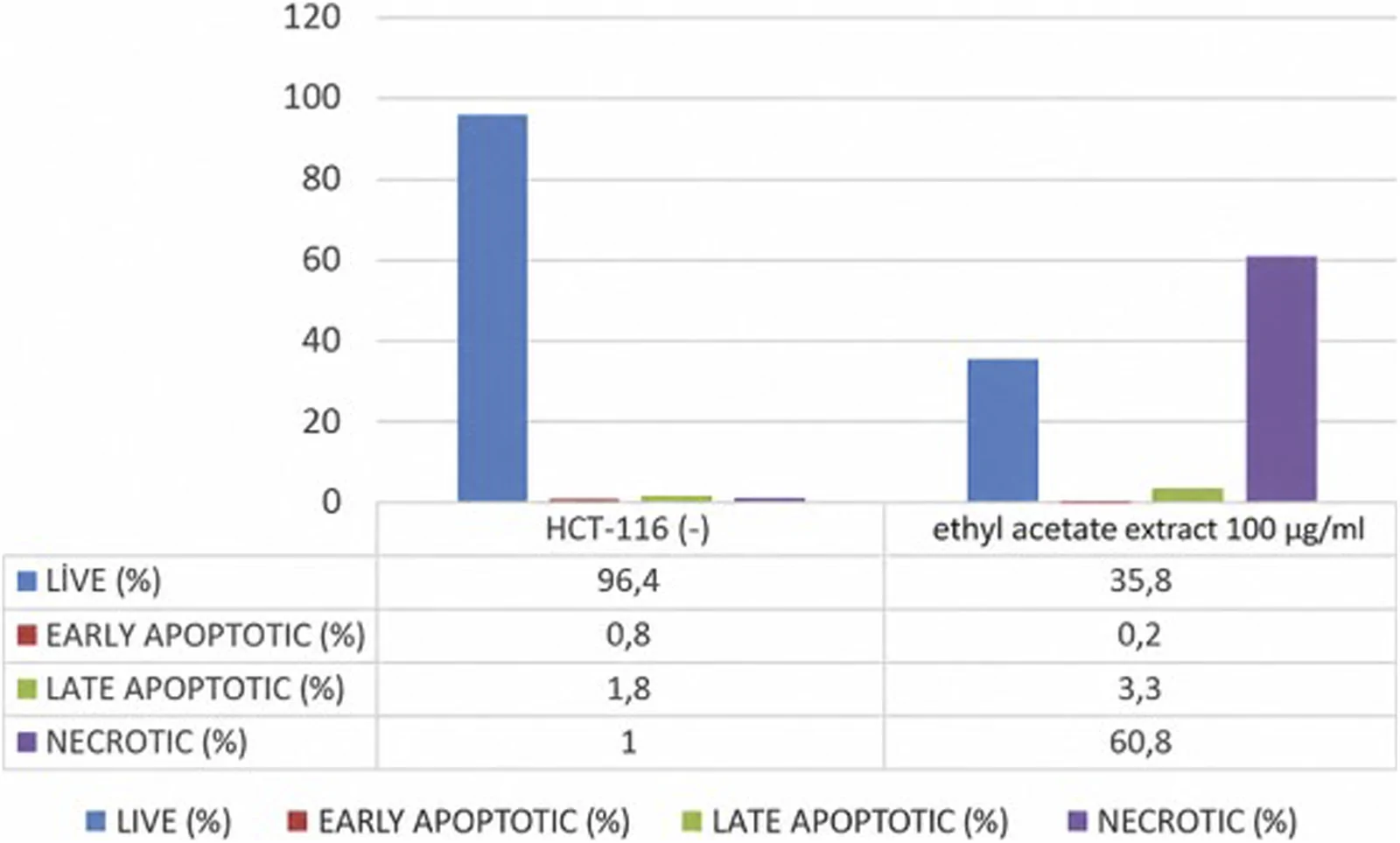
Annexin-V/PI staining results after applying ethyl acetate extract (100 µg mL^−1^) to HCT-116 cell.

**Fig. 3:**
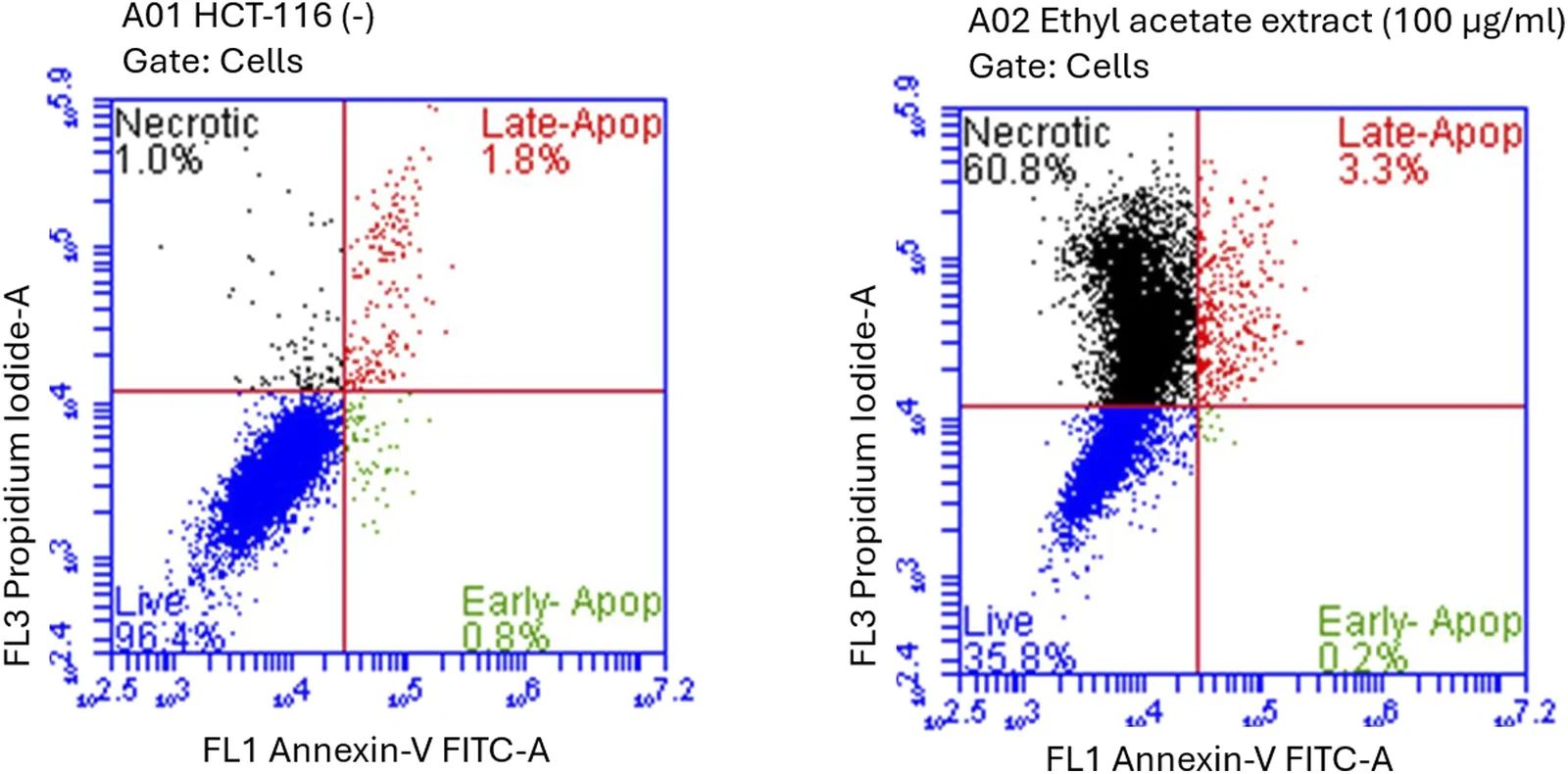
Annexin-V/PI staining results after ethyl acetate extract (100 µg mL^−1^ to HCT-116 cell. Blue: live-[(FITC-)/(PI−)]; green: early apoptotic [(FITC+)/(PI−)]; red: late apoptotic [(FITC+)/(PI+)]; black: shows necrotic [(FITC+)/(PI+)] cells.

### Validation of external QSAR models

External ChEMBL-derived QSAR models corresponding to the experimentally tested and docked targets were evaluated using Murcko scaffold split validation (Table S4). The hCA I and hCA II models showed ROC-AUC values of 0.85 and 0.91 respectively, while the AChE and BChE models reached 0.8739 and 0.8902. Their balanced accuracy values ranged from 0.74 to 0.81, with MCC values between 0.5355 and 0.6307, supporting their use as target-specific prioritization layers. The mushroom tyrosinase model also showed acceptable discrimination (ROC-AUC = 0.82), although it was interpreted more cautiously because of its weaker active-class performance. Accordingly, QSAR outputs were used as hypothesis-generating prioritization scores, together with applicability-domain assessment and docking results, rather than as substitutes for experimental IC_50_ or Ki values.

### Integrated candidate evidence ranking

The integrated evidence summary for prioritized *H. ekerii* metabolites is presented in Table S5. Myricetin was assigned to the highest-priority tier, reflecting reliable AChE-selective QSAR support, exploratory hCA-related support, and strong antioxidant structure–pattern integration. *p*-Coumaric acid was classified as a target-specific priority candidate, mainly on the basis of reliable hCA II-related support together with moderate antioxidant relevance. Ellagic acid, quercetin, and caffeic acid formed the antioxidant-priority group. Their ranking was driven by strong integrated antioxidant evidence, whereas reliable target-specific QSAR support was not observed within the evaluated enzyme modules. Apigenin was placed in an intermediate exploratory tier, combining moderate antioxidant relevance with limited hCA-related target support. The remaining EtOAc-dominant metabolites, including empetriferdinan A, fusidic acid, adlupulone, adhyperfirin, hyperfirin, and adhyperforin, were retained as exploratory target-specific candidates because their support remained limited. Vanillic acid and colupulone were placed in the lowest-priority tier under the present evidence framework. Overall, the ranking separated candidates supported by convergent target-related and antioxidant evidence from those represented by narrower or weaker support layers.

### Binding propensity and protein-ligand interaction

The docking heatmap and interaction analyses collectively demonstrated that the major phytochemicals possess favorable and target-specific binding profiles against multiple biologically relevant enzymes, particularly α-amylase and BChE (Table S6) (Fig. 4A). Among the investigated compounds, hypericin exhibited the strongest affinity toward α-amylase (−10.8 kcal mol^−1^), whereas myricetin-3-*O*-hexuronide and quercetin-3-*O*-hexoside showed the most favorable binding energies against BChE (−10.8 and −10.6 kcal mol^−1^, respectively). Notably, myricetin displayed a strong multi-target docking profile, with its most favorable interaction observed against AChE (−9.9 kcal mol^−1^), followed by α-amylase (−9.3 kcal mol^−1^), BChE (−8.8 kcal mol^−1^), and hCA II (−8.0 kcal mol^−1^). Apigenin also showed prominent affinities toward AChE and BChE (−9.3 kcal mol^−1^ for both), as well as α-amylase (−9.0 kcal mol^−1^). In contrast, *p*-coumaric acid exhibited comparatively moderate binding energies, with its most favorable score observed against AChE (−7.1 kcal mol^−1^). These interaction patterns are consistent with observed protein–ligand recognition, in which hydrogen bonding, hydrophobic interactions, and aromatic π interactions collectively govern ligand affinity and stability within enzyme active sites.^73,74^

**Fig. 4:**
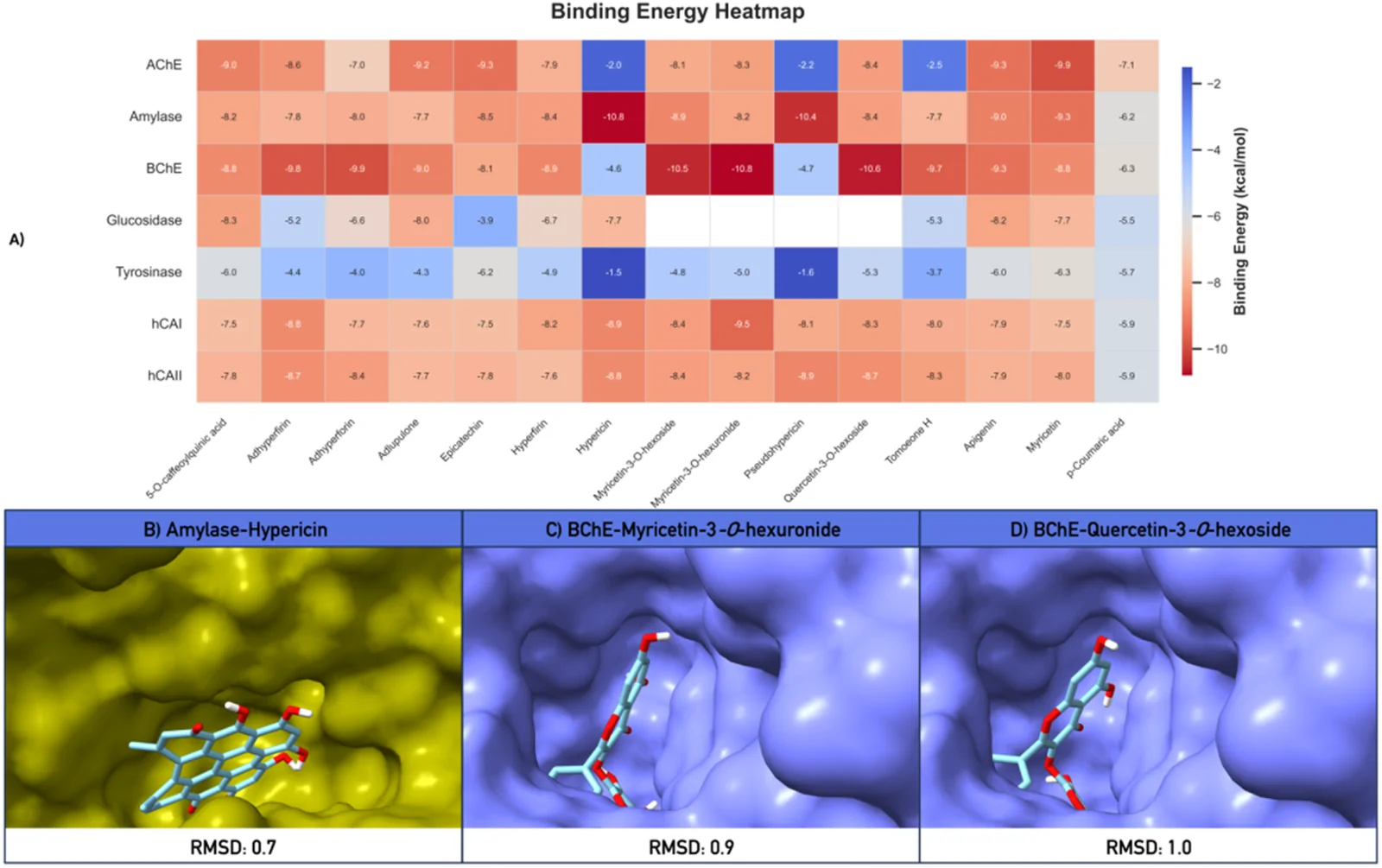
(A) Heatmap of docking binding energies (kcal mol^−1^) of major phytochemicals against selected therapeutic enzymes. (B) Binding pose of hypericin within the α-amylase active site. (C and D) Docking conformations of myricetin-3-*O*-hexuronide and quercetin-3-*O*-hexoside within the BChE catalytic gorge, illustrating favorable ligand accommodation and pocket complementarity.

Within the α-amylase catalytic pocket, hypericin adopted a compact and highly stable conformation characterized by extensive hydrophobic enclosure and polar stabilization. The ligand established hydrogen-bond interactions with key catalytic residues, including Asp197 and Glu233, while additional hydrophobic contacts with aromatic residues such as Trp59 and Leu165 promoted deep accommodation within the binding cavity (Fig. 4B). The low RMSD value (0.7 Å) further confirmed the conformational stability and reliability of the predicted docking pose.^75,76^ The large conjugated aromatic scaffold of hypericin likely enhanced π-mediated and van der Waals interactions within the hydrophobic cleft, a feature commonly associated with improved inhibitory potential against carbohydrate-hydrolyzing enzymes.^77,78^

Similarly, myricetin-3-*O*-hexuronide and quercetin-3-*O*-hexoside displayed highly favorable interaction profiles within the catalytic gorge of BChE. Myricetin-3-*O*-hexuronide formed an extensive hydrogen-bonding network involving Asp70, Gly115, Gly117, Tyr128, Ser198, Ser287, and His438, while also engaging in aromatic interactions with Phe329 **(**Fig. 4C). Quercetin-3-*O*-hexoside exhibited a comparable binding orientation, stabilized through hydrogen bonds with Asp70, Gly115, Gly117, Tyr128, Glu197, and Ser198, alongside hydrophobic and π-interactions within the enzyme gorge (Fig. 4D).^79^ In both complexes, the flavonoid aromatic cores occupied the hydrophobic channel, whereas the hydroxyl-rich glycosidic moieties remained partially solvent-exposed, enabling enhanced polar stabilization and conformational adaptability. The low RMSD values (0.9–1.0 Å) obtained for these complexes further supported the stability and reproducibility of the docking conformations.^80^ In addition to the glycosylated flavonoids, the corresponding aglycone-type metabolites also showed notable binding characteristics. Myricetin adopted a favorable pose within the AChE catalytic gorge, where its orientation was stabilized by multiple hydrogen bonds involving Gln71, Asp74, Gly120, Tyr133, Tyr337, and His447. Hydrophobic and π-stacking interactions with Trp86 further supported its compact accommodation within the active-site region. Apigenin exhibited a similarly favorable cholinesterase-binding profile. In AChE, it formed hydrogen bonds with Trp86, Tyr133, and Glu202, accompanied by hydrophobic contacts with Trp86, Phe297, and Phe338 and π-stacking with Tyr341. Its BChE complex was stabilized through hydrogen bonds with Thr120 and Glu197, hydrophobic interactions with Trp82, Tyr332, and Trp430, and aromatic stacking with Trp82. These results indicate that flavonoid aglycones, together with their glycosylated counterparts, may contribute substantially to the predicted enzyme-binding potential of *H. ekerii* metabolites. By comparison, *p*-coumaric acid showed weaker but still target–relevant interactions, including an AChE docking score of −7.1 kcal mol^−1^ and moderate hCA I/hCA II affinities (−5.9 kcal mol^−1^), suggesting a more limited structure-based contribution than the flavonoid candidates.

Overall, the observed interaction profiles suggest that these polyphenolic metabolites possess a balance of hydrophobic and polar functionalities that facilitate efficient accommodation within diverse enzymatic pockets. The most pronounced structure-based signals were observed for hypericin, myricetin-3-*O*-hexuronide, quercetin-3-*O*-hexoside, myricetin, and apigenin, which collectively showed favorable binding behavior across carbohydrate-hydrolyzing enzymes and cholinesterases. The combination of catalytic-site hydrogen bonding, hydrophobic enclosure, and aromatic stacking interactions likely underlies the strong docking affinities observed for the leading compounds. Such interaction behavior aligns with previous reports highlighting glycosylated flavonoids and polyaromatic natural products as promising scaffolds for the development of multifunctional enzyme inhibitors with potential therapeutic relevance.^81–83^

### Dynamics of the top docking complexes

#### Conformational behavior and ligand stability

RMSD analysis showed that most complexes converged rapidly and maintained stable conformational behavior throughout the 100 ns simulations (Fig. 5A). Among the investigated systems, C2 and C3 displayed the greatest structural stability, with average RMSD values of 0.21 and 0.23 Å, respectively, indicating minimal deviation from the initial docked conformations. Similarly, C4 maintained a low RMSD profile (0.38 Å), suggesting preservation of the global protein architecture during ligand binding.^84^ In contrast, C1 exhibited moderate structural adaptation with a gradual increase toward the final simulation stage, whereas C5 showed pronounced conformational changes, reaching a maximum RMSD of 7.43 Å between 40 and 80 ns before partially stabilizing. Such elevated deviations in C5 likely reflect transient ligand displacement or conformational rearrangement within the binding pocket rather than complete protein destabilization.^85^ Overall, the RMSD profiles suggest that C2, C3, and C4 formed highly stable complexes, while C5 exhibited comparatively dynamic binding behavior.^86^

**Fig. 5:**
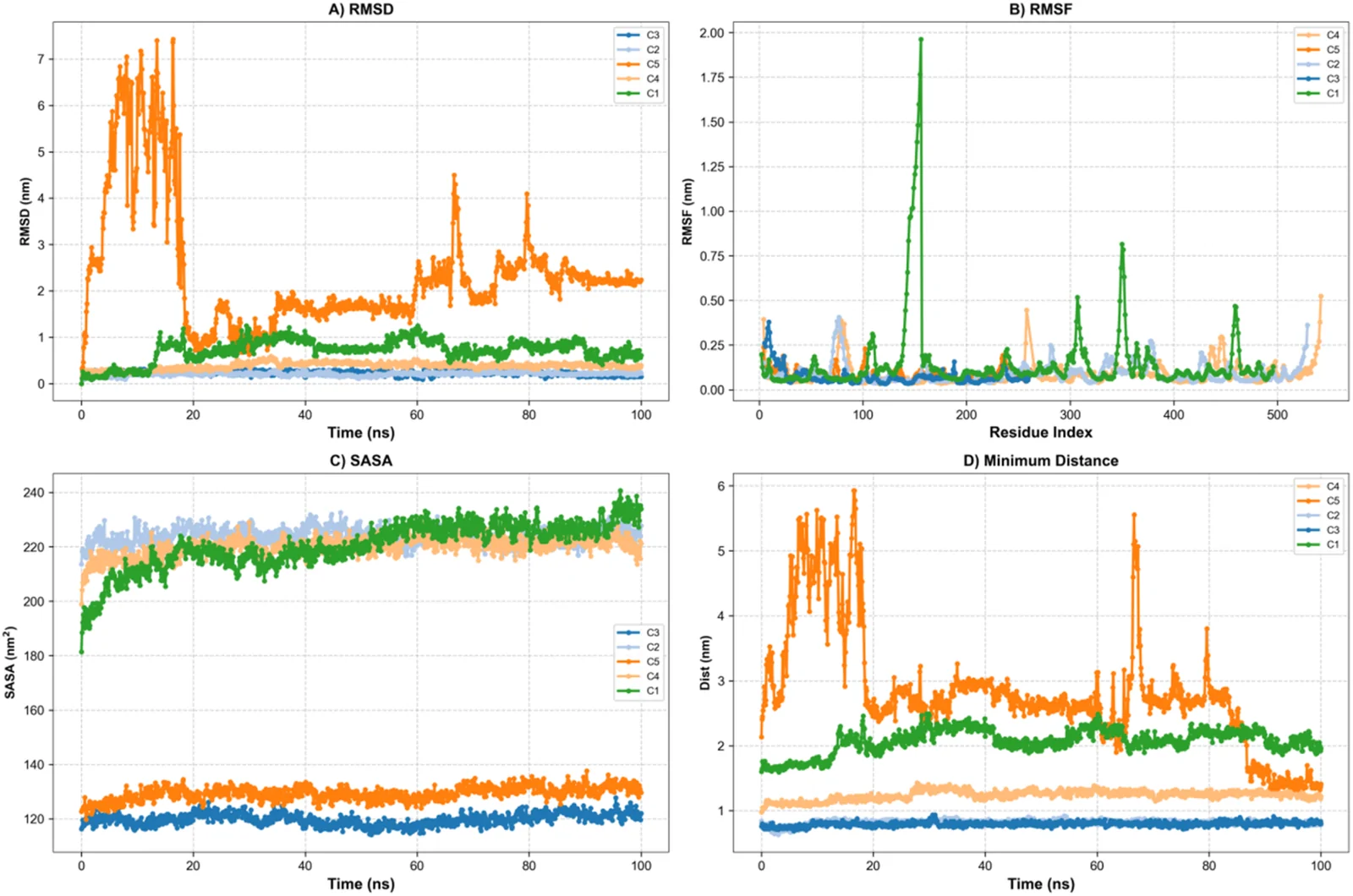
Dynamic profiles of selected docking complexes over 100 ns: (A) RMSD, (B) RMSF, (C) SASA, and (D) protein–ligand distance. The selected complexes are coded as follows: α-amylase–hypericin (C1), BChE–myricetin-3-*O*-hexuronide (C2) hCA1–myricetin-3-*O*-hexuronide, (C3) AChE–adlupulone, (C4) hCAII–pseudohypericin (C5).

#### Residual fluctuation

Residue-level flexibility analysis further supported the RMSD findings by revealing generally low backbone fluctuations across all systems (Fig. 5B). C3 and C5 exhibited the lowest average root mean square fluctuation (RMSF) values (∼0.07–0.08 Å), indicating highly rigid protein conformations despite the ligand instability observed in C5. Localized flexibility was mainly confined to loop or terminal regions, particularly residues 5–10 in C3 and residues 4, 23–24, and 102 in C5, which are known to be highly flexible.^87^ C2 and C4 also demonstrated low overall mobility,^88^ with slightly increased fluctuations around residues 75–78, 258, and 541–542, which correspond to solvent-exposed and flexible loop segments.

In contrast, C1 exhibited greater localized fluctuations, particularly around residues 152–156, where RMSF values reached 1.96 Å, suggesting regional flexibility that may contribute to the moderate conformational changes observed in the RMSD analysis. Importantly, none of the systems exhibited widespread residue instability, supporting the preservation of overall structural integrity during ligand binding.^89^

#### Surface area accessibility

Solvent accessible surface area (SASA) analysis revealed relatively stable solvent exposure profiles for all complexes (Fig. 5C), consistent with the preserved structural stability observed in the RMSD and RMSF analyses. C3 maintained the most stable SASA values throughout the simulation, fluctuating minimally around 120 nm^2^, which indicates a compact and well-equilibrated structure.^90^ C2 and C4 exhibited moderate SASA values (∼220 nm^2^) with slight increases during later simulation stages, suggesting minor conformational relaxation without substantial unfolding. C1 showed a more noticeable increase in SASA from approximately 193 to 222 nm^2^ over time, indicating partial surface exposure associated with its moderate structural adaptation. Similarly, C5 exhibited a gradual SASA increase, reaching approximately 130 nm^2^ during the final interval, which may reflect transient ligand repositioning and increased solvent accessibility within the binding region. Nevertheless, the absence of abrupt SASA shifts suggests that none of the complexes underwent significant structural disruption during the simulations.^91^

#### Protein-ligand distance

The distance-based interaction profiles confirm persistent ligand retention within the binding pockets for most systems (Fig. 5D). C2 and C3 exhibited the shortest and most stable interaction distances (∼0.80–0.82 Å) with 100% interaction occupancy below the 3.0 Å threshold, indicating exceptionally strong and stable ligand binding. C4 also maintained highly stable contacts, with an average interaction distance of 1.24 Å and uninterrupted interaction persistence throughout the simulation. Although C1 showed slightly larger interaction distances (∼2.07 Å), the ligand remained continuously associated with the binding site, suggesting stable but comparatively less compact interactions. In contrast, C5 exhibited substantial variability in interaction distance, ranging from 1.32 to 5.93 Å, with reduced interaction occupancy (78.9%), supporting the RMSD findings indicating transient ligand displacement and dynamic binding behavior.^92^ Collectively, these results demonstrate that ligand binding remained highly stable in C2–C4, whereas C5 exhibited weaker interaction persistence.

#### Hydrogen bond dynamics

Hydrogen-bond dynamics further clarified the intermolecular stabilization patterns observed in the interaction-distance analysis (Fig. 6). C2 exhibited the strongest hydrogen-bonding network, averaging 5.71 hydrogen bonds over the simulation, thereby contributing significantly to its structural stability and compact binding behavior. Similarly, C3 maintained a substantial hydrogen-bond network, with an average of 4.62 interactions, which supported its low RMSD and stable interaction-distance profile. C4 showed moderate hydrogen-bond occupancy (∼1.05 bonds), suggesting that its stability was maintained by a balance of transient hydrogen bonding and hydrophobic interactions.^93^

**Fig. 6:**
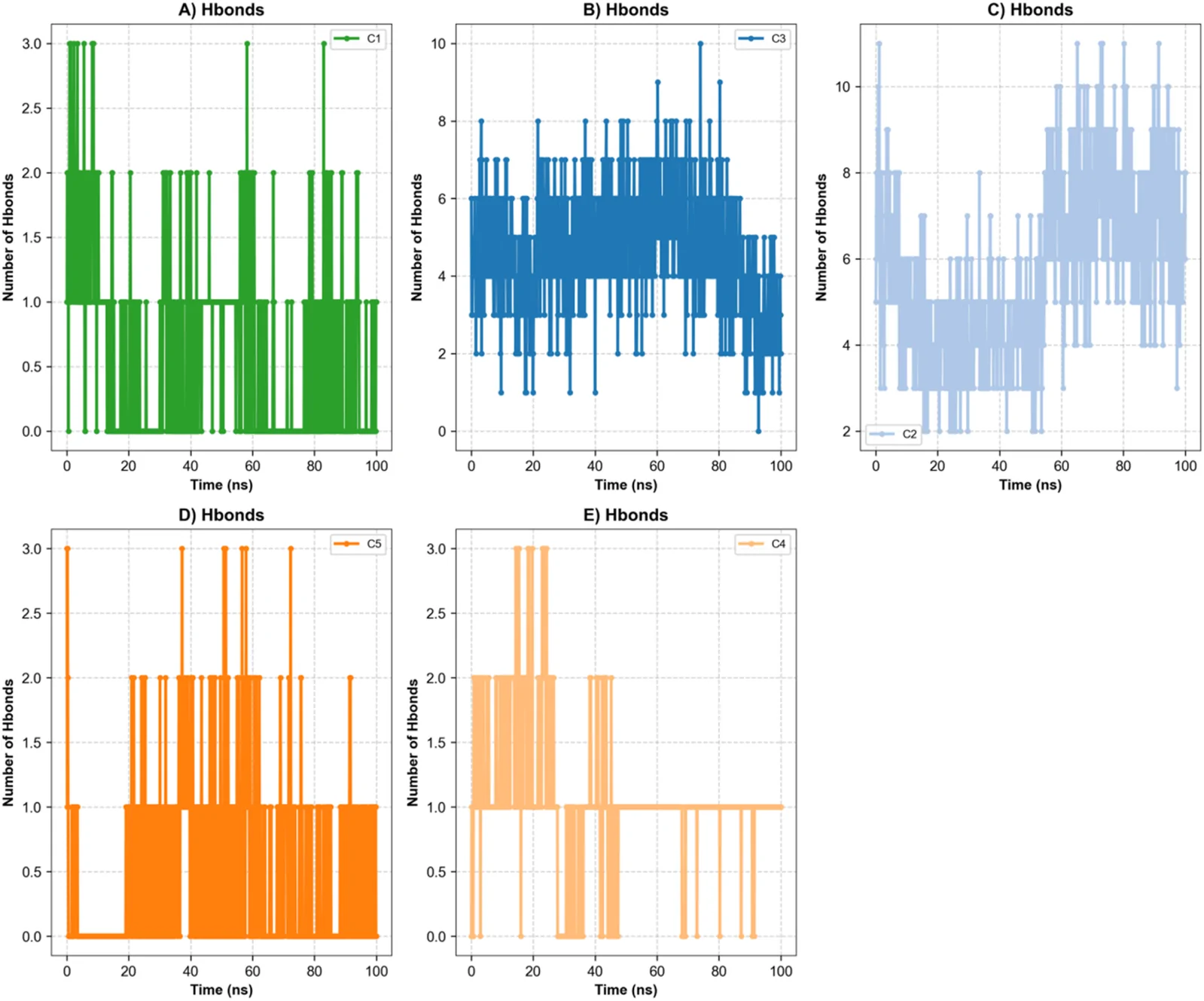
Hydrogen bond (Hbond) profiles of the selected complexes over 100 ns: (A) α-amylase–hypericin (C1), (B) BChE–myricetin-3-*O*-hexuronide (C2), (C) hCA1–myricetin-3-*O*-hexuronide (C3) (D) AChE–adlupulone (C4), (E) hCAII–pseudohypericin (C5).

In contrast, C1 and C5 displayed comparatively weak hydrogen-bonding networks, with averages of 0.62 and 0.41 hydrogen bonds, respectively. The progressive reduction in hydrogen-bond occupancy at C1 and the persistently low values at C5 likely contributed to their increased conformational flexibility and reduced interaction stability.^94^ Despite these fluctuations, transient hydrogen bonds remained sufficient to preserve ligand association throughout most of the simulations, particularly in C1. Overall, the hydrogen bond analysis highlights C2 and C3 as the most strongly stabilized complexes among the investigated systems.

## Conclusion

The present study provides the first integrated phytochemical and biological characterization of *H. ekerii*. The species was shown to contain a chemically diverse metabolite profile, including phenolic acids, flavonoids, xanthones, anthraquinones, and acylphloroglucinols. Among the tested extracts, the hydroacetone fraction was particularly prominent in terms of phenolic/flavonoid content, radical-scavenging capacity, reducing power, and inhibition of AChE, tyrosinase, and hCA II, whereas the EtOAc extract showed stronger metal-chelating activity and greater inhibition of BChE and hCA I. Under the conditions tested, *H. ekerii* extracts exhibited limited cytotoxicity and no meaningful cancer-cell selectivity. The follow-up analysis of the EtOAc/HCT-116 pair documents cellular injury near the measured IC_50_, predominantly involving a PI-positive membrane-compromised population, and should be interpreted as exploratory cell-death phenotyping rather than evidence of selective pro-apoptotic anticancer activity. Structure-based analyses further highlighted favorable target interactions for selected metabolites, especially hypericin, myricetin-3-*O*-hexuronide, quercetin-3-*O*-hexoside, myricetin, and apigenin, with molecular dynamics simulations supporting the stability of representative complexes. The QSAR-informed prioritization placed myricetin at the top of the integrated ranking, identified *p*-coumaric acid as a target-specific priority candidate, and highlighted ellagic acid, quercetin, and caffeic acid as antioxidant-priority metabolites. Overall, *H. ekerii* appears to be a promising source of antioxidant and enzyme-inhibitory constituents, while the prioritized metabolites provide a focused basis for future isolation studies and compound-level biological validation.

## Author contributions

Gokhan Zengin: investigation, methodology, formal analysis, writing – original draft, writing – review and editing. Sakina Yagi: investigation, methodology, visualization, formal analysis, writing – original draft, writing – review and editing. Mehmet Veysi Cetiz: investigation, methodology, visualization, formal analysis. Abdullahi Ibrahim Uba: investigation, methodology, visualization, formal analysis. Filip Nikolić: investigation, methodology, writing – original draft, writing – review and editing. Uroš Gašić: investigation, methodology, writing – original draft, writing – review and editing. Mehmet Maruf Balos: investigation, methodology, resources. Ahmet Erharman: investigation, methodology, writing – original draft, writing – review and editing. Nurettin Dogan: investigation, methodology, writing – original draft, writing – review and editing. Ismail Koyuncu: investigation, methodology, resources. Ismail Yapıcı: investigation, methodology, visualization, formal analysis. Ilhami Gulcin: investigation, methodology, formal analysis, writing – original draft, writing – review and editing.

## Conflicts of interest

There are no conflict to declare.

## Supplementary Material

RA-016-D6RA04698C-s001

## Data Availability

Data will be requested from authors. Supplementary information (SI): Table S1: Docking grid center and size. Table S2: LC-MS data on metabolites tentatively annotated in *Hypericum ekerii* samples (*t*_R_ – retention time (min); *Δ* ppm – mean mass accuracy error; NA – not available). Table S3: Peak areas of tentatively annotated compounds; values were obtained from full scan MS spectra. Table S4: Scaffold split performance of external ChEMBL-based QSAR models used in the prioritization. Table S5: Integrated candidate evidence summary for prioritized *Hypericum ekerii* metabolites. Table S6: The docking score (kcal mol^−1^) and interacting residues of the enzyme. Fig. S1: Chemical structures of the key bioactive compounds identified in the plant extracts, categorized by their respective compound classes: naphthodianthrones (hypericin), flavonoid glycosides (myricetin-3-*O*-hexuronide, quercetin-3-*O*-hexoside), and flavonol-myricetin, and flavone apigenin). Fig. S2: Base peak chromatograms of *Hypericum* samples obtained with different extraction solvents. Fig. S3: Heatmap of the scaled data of untargeted metabolite analysis, with the samples (both columns and rows) arranged according to the HCA (Pearson method of cluster agglomeration). Intensity of red colour indicates the abundance of the compounds in samples, as indicated in the colour scale. Fig. S4: hCA I Inhibition graph of *H. ekerii* extracts; EtOAc, Ethylacetate, ACE, acetone. Results were expressed as µg mL^−1^. Fig. S5: hCA II inhibition graph of *H. ekerii extracts;* EtOAc, Ethylacetate, ACE, acetone. Results were expressed as µg mL^−1^. See DOI: https://doi.org/10.1039/d6ra04698c.
