# Supplementary material for: Chemical profiling, biological evaluation, and QSAR-informed metabolite prioritization of Hypericum ekerii: a first report on antioxidant, enzyme inhibitory, and cytotoxic activities
Source: RSC Adv. 2026 Sep 2;16(43):52607–23. doi: 10.1039/d6ra04698c (PMC13537189; doi:10.1039/d6ra04698c)
Supplement: Supplementary file 1 [file RA-016-D6RA04698C-s001.pdf]

Supplementary Information (SI) for RSC Advances.
This journal is © The Royal Society of Chemistry 2026

Determination of Antioxidant and Enzyme Inhibitory Effects

Antioxidant (DPPH and ABTS radical scavenging, reducing power (CUPRAC and FRAP), phosphomolybdenum and metal chelating (ferrozine method)) and enzyme inhibitory activities (cholinesterase (Elmann's method), tyrosinase (dopachrome method), α-amylase (iodine/potassium iodide method), α -glucosidase (chromogenic PNPG method) and pancreatic lipase (p-nitrophenyl butyrate (p-NPB) method) were determined.

For the DPPH (1,1-diphenyl-2-picrylhydrazyl) radical scavenging assay: Sample solution was added to 4 mL of a 0.004% methanol solution of DPPH. The sample absorbance was read at 517 nm after a 30 min incubation at room temperature in the dark. DPPH radical scavenging activity was expressed as milligrams of trolox equivalents (mg TE/g extract) [1].

For ABTS (2,2′-azino-bis(3-ethylbenzothiazoline) 6-sulfonic acid) radical scavenging assay: Briefly, ABTS+ was produced directly by reacting 7 mM ABTS solution with 2.45 mM potassium persulfate and allowing the mixture to stand for 12–16 in the dark at room temperature. Prior to beginning the assay, ABTS solution was diluted with methanol to an absorbance of 0.700 ± 0.02 at 734 nm. Sample solution was added to ABTS solution (2 mL) and mixed. The sample absorbance was read at 734 nm after a 30 min incubation at room temperature. The ABTS radical scavenging activity was expressed as milligrams of trolox equivalents (mg TE/g extract) [2].

For CUPRAC (cupric ion reducing activity) activity assay: Sample solution was added to premixed reaction mixture containing CuCl2 (1 mL, 10 mM), neocuproine (1 mL, 7.5 mM) and NH4Ac buffer (1 mL, 1 M, pH 7.0). Similarly, a blank was prepared by adding sample solution (0.5 mL) to premixed reaction mixture (3 mL) without CuCl2 . Then, the sample and blank absorbances were read at 450 nm after a 30 min incubation at room temperature. The absorbance of the blank was subtracted from that of the sample. CUPRAC activity was expressed as milligrams of trolox equivalents (mg TE/g extract) [3].

For FRAP (ferric reducing antioxidant power) activity assay: Sample solution was added to premixed FRAP reagent (2 mL) containing acetate buffer (0.3 M, pH 3.6), 2,4,6-tris(2-pyridyl)-S-triazine (TPTZ) (10 mM) in 40 mM HCl and ferric chloride (20 mM) in a ratio of 10:1:1 (v/v/v). Then, the sample absorbance was read at 593 nm after a 30 min incubation at room temperature. FRAP activity was expressed as milligrams of trolox equivalents (mg TE/g extract) [4].

For phosphomolybdenum method: Sample solution was combined with 3 mL of reagent solution (0.6 M sulfuric acid, 28 mM sodium phosphate and 4 mM ammonium molybdate). The sample absorbance was read at 695 nm after a 90 min incubation at 95 °C. The total antioxidant capacity was expressed as millimoles of trolox equivalents (mmol TE/g extract) [5].

For metal chelating activity assay: Briefly, sample solution was added to FeCl2 solution (0.05 mL, 2 mM). The reaction was initiated by the addition of 5 mM ferrozine (0.2 mL). Similarly, a blank was prepared by adding sample solution (2 mL) to FeCl2 solution (0.05 mL, 2 mM) and water (0.2 mL) without ferrozine. Then, the sample and blank absorbances were read at 562 nm after 10 min incubation at room temperature. The absorbance of the blank was subtracted from that of the sample. The metal chelating activity was expressed as milligrams of EDTA (disodium edetate) equivalents (mg EDTAE/g extract) [6].

For Cholinesterase (ChE) inhibitory activity assay: Sample solution (was mixed with DTNB (5,5-dithio-bis(2-nitrobenzoic) acid, Sigma, St. Louis, MO, USA) (125 µL) and AChE (acetylcholinesterase (Electric ell acetylcholinesterase, Type-VI-S, EC 3.1.1.7,Sigma)), or BChE (butyrylcholinesterase (horse serum butyrylcholinesterase, EC 3.1.1.8, Sigma)) solution (25 µL) in Tris–HCl buffer (pH 8.0) in a 96-well microplate and incubated for 15 min at 25 °C. The reaction was then initiated with the addition of acetylthiocholine iodide (ATCI, Sigma) or butyrylthiocholine chloride (BTCl, Sigma) (25 µL). Similarly, a blank was prepared by adding sample solution to all reaction reagents without enzyme (AChE or BChE) solution. The sample and blank absorbances were read at 405 nm after 10 min incubation at 25 °C. The absorbance of the blank was subtracted from that of the sample and the cholinesterase inhibitory activity was expressed as galanthamine equivalents (mg GALAE/g extract) [7].

For Tyrosinase inhibitory activity assay: Sample solution was mixed with tyrosinase solution (40 µL, Sigma) and phosphate buffer (100 µL, pH 6.8) in a 96-well microplate and incubated for 15 min at 25 °C. The reaction was then initiated with the addition of L-DOPA (40 µL, Sigma). Similarly, a blank was prepared by adding sample solution to all reaction reagents without enzyme (tyrosinase) solution. The sample and blank absorbances were read at 492 nm after a 10 min incubation at 25 °C. The absorbance of the blank was subtracted from that of the sample and the tyrosinase inhibitory activity was expressed as kojic acid equivalents (mgKAE/g extract) [8].

For α-amylase inhibitory activity assay: Sample solution was mixed with α-amylase solution (ex-porcine pancreas, EC 3.2.1.1, Sigma) (50 µL) in phosphate buffer (pH 6.9 with 6 mM sodium chloride) in a 96-well microplate and incubated for 10 min at 37 °C. After pre-incubation, the reaction was initiated with the addition of starch solution (50 µL, 0.05%). Similarly, a blank was prepared by adding sample solution to all reaction reagents without enzyme (α-amylase) solution. The reaction mixture was incubated 10 min at 37 °C. The reaction was then stopped with the addition of HCl (25 µL, 1 M). This was followed by addition of the iodine-potassium iodide solution (100 µL). The sample and blank absorbances were read at 630 nm. The absorbance of the blank was subtracted from that of the sample and the α-amylase inhibitory activity was expressed as acarbose equivalents (mmol ACE/g extract) [9].

For α-glucosidase inhibitory activity assay: Sample solution was mixed with glutathione (50 µL), α-glucosidase solution (from Saccharomyces cerevisiae, EC 3.2.1.20, Sigma) (50 µL) in phosphate buffer (pH 6.8) and PNPG (4-N-trophenyl-α-D-glucopyranoside, Sigma) (50 µL) in a 96-well microplate and incubated for 15 min at 37 °C. Similarly, a blank was prepared by adding sample solution to all reaction reagents without enzyme (α-glucosidase) solution. The reaction was then stopped with the addition of sodium carbonate (50 µL, 0.2 M). The sample and blank absorbances were read at 400 nm. The absorbance of the blank was subtracted from that of the sample and the α-glucosidase inhibitory activity was expressed as acarbose equivalents (mmol ACE/g extract) [10].

Human carbonic anhydrase enzyme was purified by Sepharose 4 B-L-tyrosine sulfanilamide affinity column and characterized by SDS-PAGE as previously applied [11]. The esterase activity of the carbonic anhydrase (CA) enzyme yields a yellow-colored aromatic p-nitrophenolate compound at a pH of around pH=7.5. By taking advantage of this transformation, the esterase activity of CA enzymes can be determined spectrophotometrically(Tel et al., 2025a). Various concentrations of the samples in 0.05 M pH=7.4 Tris-SO_4, 0.07 mM of p-nitrophenylacetate (in 1:25 acetone: water), and 20 µL of the enzyme were gently vortexed, and as soon the as the addition of the enzyme, the absorbance change at 348 nm was monitored by measuring minute intervals for three minutes. Control reactions and blank reactions were set up without inhibitors and enzymes. These steps were repeated until more than half of the enzyme activity was inhibited. All data were processed, and graphs were created using GraphPad Prism 8.0.2. IC_{50} value refers in this study to the concentration of an inhibitor needed to inhibit an enzyme activity response by 50%. The calculation of the IC_{50} values was performed using the GraphPad Prism 8.4.0 non-linear regression-[inhibitor]-normalized response (y values 100 down to 0) model.

Table S1. Docking grid center and size

Target Protein	PDB ID	Center X, Y, Z (Å)	Grid Size X, Y, Z (Å)
AChE	7E3H	-54.44, 32.90, -28.65	25, 25, 25
BChE	6EQP	32.16, -16.33, 40.73	25, 25, 25
α-mylase	2QV4	14.188, 48.964, 22.886	28, 28, 24
α-glucosidase	7KBJ	-18.79, 2.18, 15.75	25, 25, 25
Tyrosinase	6QXD	21.81, 12.22, 91.40	25, 25, 25
hCAI	3LXE	-19.049, 36.885, 44.812	40, 40, 40
hCAII	4IWZ	14.059, 4.757, 14.338	40, 40, 40

Table S2. LC-MS data on metabolites tentatively annotated in *Hypericum ekerii* samples (t_R – retention time (min); Δ ppm – mean mass accuracy error; NA – not available).

No	Compound name	t_R, min	Molecular formula, [M–H]–	Calculated mass, *m/z*	Exact mass, *m/z*	Δ ppm	MS² Fragments, (% Base Peak)	Level of identification	Previously reported in *Hypericum*
Hydroxybenzoic acids									
**1**	**Hydroxybenzoic acid isomer**	0.58	$C_7H_5O_3^-$	137.02442	137.02433	0.68	**93.03455**(100), 137.02428(34)	1	[12]
**2**	**Gallic acid hexoside**	0.60	$C_{13}H_{15}O_{10}^-$	331.06707	331.06683	0.72	125.02438(57), 151.00368(24), 168.00641(66), 169.01413(64), 313.05664(15), **331.06702**(100)	2	[13]
**3**	**Hydroxybenzoic acid hexoside**	0.61	$C_{13}H_{15}O_8^-$	299.07724	299.07701	0.77	93.03452(37), **137.02432**(100)	2	NA
**4**	**Gallic acid**	0.63	$C_7H_5O_5^-$	169.01425	169.01414	0.61	**125.02441**(100), 169.01414(43)	1	[14]
**5**	**Vanillic acid hexoside**	0.64	$C_{14}H_{17}O_9^-$	329.08781	329.08761	0.60	108.02164(14), 123.04514(36), 152.01146(18), **167.03491**(100), 329.08801(13)	2	[15]
**6**	**Dihydroxybenzoic acid hexoside**	0.66	$C_{13}H_{15}O_9^-$	315.07216	315.07200	0.49	108.02160(41), 109.02941(33), **152.01141**(100), 153.01918(59), 315.07202(50)	2	[16]
**7**	**3,4-Dihydroxybenzoic acid (Protocatechuic acid)**	0.82	$C_7H_5O_4^-$	153.01930	153.01923	0.48	**109.02950**(100), 153.01932(30)	1	[14]
**8**	**Ellagic acid**	5.06	$C_{14}H_5O_8^-$	300.99899	300.99883	0.53	300.99869(100)	1	[12]
**9**	**Vanillic acid**	5.13	$C_8H_7O_4^-$	167.03498	167.03487	0.66	**108.02171**(100), 123.04540(10), 152.01059(39), 167.03519(29)	1	[12]
**10**	**p-Hydroxybenzoic acid**	5.52	$C_7H_5O_3^-$	137.02442	137.02437	0.33	**93.03452**(100), 137.02438(57)	1	[12]
Hydroxycinnamic acids									

**11**	**Caffeoylquinic acid hexoside**	0.64	$C_{22}H_{27}O_{14}^{-}$	515.14063	515.14023	0.77	135.04507(22), 161.02434(38), 173.04543(18), **179.03488**(100), 191.05598(61), 335.07690(29)	2	NA
**12**	**Caffeic acid hexoside**	0.71	$C_{15}H_{17}O_{9}^{-}$	341.08781	341.08726	1.60	135.04514(23), **179.03491**(100)	2	[17]
**13**	**5-O-Caffeoylquinic acid**	0.74	$C_{16}H_{17}O_{9}^{-}$	353.08781	353.08699	2.30	135.04512(23), 161.02449(4), 173.04535(4), 179.03491(67), **191.05603**(100)	1	[18]
**14**	**5-O-Coumaroylquinic acid**	1.11	$C_{16}H_{17}O_{8}^{-}$	337.09289	337.09275	0.42	119.05021(31), 155.03505(3), **163.04005**(100), 173.04535(3), 191.05606(14)	2	[18]
**15**	**p-Coumaric acid hexoside**	1.37	$C_{15}H_{17}O_{8}^{-}$	325.09289	325.09270	0.60	119.05019(53), 145.02948(21), **163.04004**(100)	2	[19]
**16**	**Caffeic acid**	2.05	$C_{9}H_{7}O_{4}^{-}$	179.03498	179.03489	0.50	**135.04512**(100), 179.03506(21)	1	[20]
**17**	**p-Coumaric acid**	4.36	$C_{9}H_{7}O_{3}^{-}$	163.04007	163.03998	0.56	**119.05022**(100), 163.03983(14)	1	[12]
**18**	**Caffeoylshikimic acid**	4.47	$C_{16}H_{15}O_{8}^{-}$	335.07724	335.07697	0.80	135.04506(21), **161.02431**(100), 179.03488(8)	2	NA
Flavan-3-ols									
**19**	**Procyanidin**	0.75	$C_{30}H_{25}O_{13}^{-}$	593.13007	593.12920	1.47	125.02437(89), 137.02440(16), 177.01927(98), 245.08171(30), 289.07156(82), **407.07672**(100)	2	[21]
**20**	**(Epi)gallocatechin**	0.85	$C_{15}H_{13}O_{7}^{-}$	305.06668	305.06650	0.57	**125.02438**(100), 137.02435(18), 165.01945(24), 167.03471(29), 179.03494(19), 219.06596(22)	2	[22]
**21**	**B type procyanidin dimer 1**	1.79	$C_{30}H_{25}O_{12}^{-}$	577.13515	577.13455	1.03	203.07135(8), 205.05061(10), 245.08160(36), 273.04001(8) 289.07166(97), **407.07684**(100)	2	[18]
**22**	**(Epi)catechin**	2.77	$C_{15}H_{13}O_{6}^{-}$	289.07176	289.07157	0.66	109.02946(77), 125.02441(77), 151.04005(41), 203.07126(80), 205.05057(53), **245.08189**(100)	1	[22]
**23**	**B type procyanidin dimer 2**	5.32	$C_{30}H_{25}O_{12}^{-}$	577.13515	577.13390	2.17	**125.02437**(100), 137.02428(16), 161.02441(23), 245.08136(23), 289.07159(59), 407.07666(39)	2	[18]
Flavonoid C-glycosides									

**24**	**Apigenin 6,8-di-O-hexoside**	4.10	C27H29O15 –	593.15119	593.15102	0.29	**353.06577**(100), 365.06543(4), 383.07645(63), 413.08698(8), 473.10834(45), 503.11880(15)	2	[23]
**25**	**Luteolin 8-C-hexoside-6-C-pentoside**	4.75	C26H27O15 –	579.13554	579.13493	1.06	309.03967(52), 327.05060(46), 339.05042(19), 357.06085(47), 429.08215(21), **459.09277**(100)	2	NA
**26**	**Luteolin 8-C-glucoside**	4.84	C21H19O11 –	447.09329	447.09254	1.68	299.05566(6), **327.05075**(100), 357.06100(30)	1	[24]
**27**	**Chrysin 6,8-di-C-hexoside**	4.99	C27H29O14 –	577.15628	577.15546	1.42	**337.07156**(100), 367.08151(61), 397.09277(6), 407.07703(10), 457.11356(59), 487.12396(24)	2	NA
**28**	**Apigenin C-hexoside-C-pentoside (Iso)schaftoside**	5.09	C26H27O14 –	563.14063	563.14020	0.77	**293.04538**(100), 311.05585(21), 323.05612(10), 327.05231(12), 341.06610(14), 413.08807(27)	2	NA
**29**	**Apigenin 8-C-glucoside**	5.12	C21H19O10 –	431.09837	431.09754	1.94	283.06104(17), **311.05591**(100), 341.06598(9)	1	[25]
**30**	**Luteolin 8-C-(6"-malonyl)-hexoside**	5.26	C24H21O14 –	533.09368	533.09270	1.84	297.04019(8), 299.05643(6), **327.05081**(100), 357.06104(20)	2	NA
Flavonoid O-glycosides									
**31**	**Myricetin 3-O-hexoside**	4.70	C21H19O13 –	479.08311	479.08238	1.54	178.99884(2), 271.02444(2), **316.02228**(100), 317.02991(13), 479.08298(4)	2	[21]
**32**	**Myricetin 3-O-(6"-rhamnosyl)-hexoside**	4.72	C27H29O17 –	625.14102	625.14043	0.95	178.99860(3), **316.02225**(100), 317.02948(16), 625.14050(13)	2	[26]
**33**	**Quercetin 3-O-rhamnoside-7-O-hexuronide**	4.78	C27H27O17 –	623.12537	623.12514	0.37	151.00383(4), 178.99869(2), **301.03506**(100), 447.09314(34)	2	NA
**34**	**Myricetin 3-O-hexuronide**	4.78	C21H17O14 –	493.06238	493.06162	1.54	151.00354(7), 178.99846(15), 316.02267(2), **317.03000**(100)	2	[27]
**35**	**Quercetin 3-O-rhamnoside-7-O-hexoside**	4.82	C27H29O16 –	609.14611	609.14553	0.95	299.01944(60), 300.02686(10), 301.03503(51), **446.08481**(100), 447.09204(26), 463.08755(12)	2	NA
**36**	**Quercetin 3,7-di-O-hexoside**	4.90	C27H29O17 –	625.14102	625.14048	0.86	151.00349(3), 178.99832(3), **300.02737**(100), 301.03497(56), 463.08783(7), 625.13971(13)	2	[28]
**37**	**Quercetin 3-O-rhamoside-7-O-pentoside**	4.93	C26H27O15 –	579.13554	579.13541	0.22	299.01974(59), 300.02808(7), 301.03601(31), 433.07739(7), **446.08475**(100),	2	NA

							447.09167(14)		
**38**	**Myricetin 3-O-pentoside**	5.08	C20H17O12–	449.07255	449.07188	1.49	178.99883(2), **316.02234**(100), 317.02902(7)	2	NA
**39**	**Quercetin 3-O-(2"-hexosyl)-hexuronide**	5.08	C27H27O18–	639.12029	639.11970	0.92	151.00349(4), 178.99995(2), **301.03519**(100), 301.07968(3)	2	NA
**40**	**Quercetin 3-O-(6"-rhamnosyl)-glucoside (Rutin)**	5.16	C27H29O16–	609.14611	609.14546	1.06	151.00359(4), 178.99861(3), 300.02737(81), **301.03220**(100)	1	18
**41**	**Quercetin 3-O-glucoside**	5.20	C21H19O12–	463.08820	463.08754	1.42	151.00374(2), 178.9986(2), 255.02904(2), 271.02441(3), **300.02737**(100), 301.03506(36)	1	18
**42**	**Quercetin 3-O-hexuronide**	5.27	C21H17O13–	477.06746	477.06673	1.55	151.00371(9), 178.99875(9), 273.04004(2), 283.02417(2), **301.03522**(100)	2	14
**43**	**Quercetin 3-O-(6''-acetyl)-hexoside**	5.45	C23H21O13–	505.09877	505.09812	1.27	151.00369(3), 178.99857(2), **300.02725**(100), 301.03491(29), 505.09592(3)	2	13
**44**	**Kaempferol 3-O-(6"-rhamnosyl)-hexoside**	5.47	C27H29O15–	593.15119	593.15083	0.61	151.00385(2), 178.99864(1), 255.03006(2), 257.04507(2), 284.03247(73), **285.04019**(100)	2	29
**45**	**Kaempferol 3-O-glucoside**	5.47	C21H19O11–	447.09329	447.09265	1.43	151.00383(2), 227.03476(3), 255.02988(11), **284.03241**(100), 285.04013(23), 447.09320(22)	1	30
**46**	**Quercetin 3-O-pentoside**	5.48	C20H17O11–	433.07764	433.07705	1.35	151.00415(2), 178.9986(2), 271.0603(23), **300.02734**(100), 301.03497(22)	2	18
**47**	**Quercetin 3-O-rhamnoside**	5.58	C21H19O11–	447.09329	447.09274	1.22	151.00372(5), 178.99858(4), **300.02734**(100), 301.03506(66), 447.09253(21)	1	31
**48**	**Kaempferol 3-O-hexuronide**	5.59	C21H17O12–	461.07255	461.07211	0.95	113.02430(8), 229.05045(4), 257.04535(5), **285.04013**(100)	2	27
**49**	**Kaempferol 3-O-pentoside**	5.71	C20H17O10–	417.08272	417.08227	1.08	227.03465(3), 255.02988(9), **284.03238**(100), 285.04004(15), 417.08221(16)	2	32
**50**	**Eriodictyol 7-O-hexoside**	5.78	C21H21O11–	449.10894	449.10820	1.63	135.04507(25), **151.00372**(100), 241.05054(47), 287.05585(80)	2	33
**51**	**Kaempferol 3-O-(6"-malonyl)-hexoside**	5.79	C24H21O14–	533.09368	533.09327	0.76	255.03011(3), 257.04520(2), **284.03241**(100), 285.04016(83), 489.10239(2)	2	26
**52**	**Kaempferol 3-O-(6''-acetyl)-hexoside**	5.79	C23H21O12–	489.10385	489.10341	0.90	229.05040(2), 255.02977(5), **284.03244**(100), 285.04019(69)	2	34

**53**	**Myricetin 3-O-(6''-p-coumaroyl)-hexoside**	5.86	C30H25O15–	625.11989	625.11931	0.94	151.00410(2), 178.99858(3), **316.02234**(100), 317.02954(15), 463.08737(5), 479.08276(14)	2	NA
**54**	**Quercetin 3-O-(2''-acetyl)-hexuronide**	6.01	C23H19O14–	519.07803	519.07757	0.89	151.00366(9), 178.99861(9), 300.02744(28), **301.03513**(100), 459.05692(5)	2	35
**55**	**Kaempferol 3-O-rhamnoside**	6.06	C21H19O10–	431.09837	431.09773	1.50	284.03250(38), **285.04019**(100), 431.0979(56)	2	14
**56**	**Quercetin 3-O-(2''-p-coumaroyl)-hexoside**	6.15	C30H25O14–	609.12498	609.12436	1.02	145.02925(2), 151.00371(3), 178.99855(3), **300.02728**(100), 301.03488(51), 463.08783(32)	2	24
Flavonoid aglycones									
**57**	**Myricetin**	5.86	C15H9O8–	317.03029	317.03017	0.38	137.02441(46), 151.00365(92), 165.01930(10), **178.99858**(100), 193.01411(11), 317.03000(99)	1	12
**58**	**Luteolin**	6.41	C15H9O6–	285.04046	285.04032	0.50	151.00375(2), 175.04068(2), 199.04060(2), 241.05025(2), **285.04025**(100)	1	18
**59**	**Quercetin**	6.42	C15H9O7–	301.03538	301.03527	0.34	107.01393(6), 121.02953(18), **151.00363**(100), 178.99852(52), 273.04074(10), 301.03522(78)	1	18
**60**	**Apigenin**	6.85	C15H9O5–	269.04555	269.04541	0.52	149.02437(3), 151.00369(3), 225.05553(2), **269.04523**(100)	1	30
**61**	**Quercetin 3,7-dimethyl ether**	6.90	C17H13O7–	329.06668	329.06647	0.64	271.02512(2), 285.04077(5), 299.01947(63), **314.04294**(100), 329.06689(34)	2	36
**62**	**Kaempferol**	6.92	C15H9O6–	285.04046	285.04026	0.72	151.00369(2), 257.04529(3), **285.04022**(100)	1	18
**63**	**Chrysoeriol**	6.94	C16H11O6–	299.05611	299.05587	0.81	256.03772(2), **284.03244**(100), 299.05618(32)	1	37
Xanthones									
**64**	**Mangiferin**	4.91	C19H17O11–	421.07764	421.07696	1.61	259.06104(45), 259.11536(9), 271.02377(10), 301.03534(81), 331.04541(14), **421.18573**(100)	1	31
**65**	**Dihydroxydimethoxyxanthone 1**	6.37	C15H11O6–	287.05611	287.05581	1.04	257.00916(17), **272.03253**(100), 287.05600(20)	2	14
**66**	**Hydroxydimethoxyxanthone**	6.93	C15H11O5–	271.06120	271.06108	0.41	228.04265(4), 241.01372(4), **256.03751**(100), 271.06104(18)	2	38

**67**	**Trihydroxydimethoxyxanthone**	7.25	C15H11O7–	303.05103	303.05085	0.57	273.00400(11), **288.02740**(100), 303.05099(26)	2	39
**68**	**Dihydroxydimethoxyxanthone 2**	7.86	C15H11O6–	287.05611	287.05598	0.46	257.00900(39), **272.03235**(100), 287.05603(9)	2	14
**69**	**Dihydroxytrimethoxyxanthone**	8.02	C16H13O7–	317.06668	317.06651	0.53	259.02505(3), 287.01956(47), **302.04288**(100), 317.06702(7)	2	40
Anthraquinones									
**70**	**5,ω-Dihydroxyemodin**	6.23	C15H9O7–	301.03538	301.03524	0.44	**165.01927**(100), 257.04568(7), 301.03513(3)	2	31
**71**	**Skyrin hexoside**	6.46	C36H27O15–	699.13554	699.13498	0.81	**151.00363**(100), 385.07117(50), 443.04041(41), 537.08270(56)	2	31
**72**	**Skyrin**	7.14	C30H17O10–	537.08272	537.08215	1.07	107.01378(12), **151.00357**(100), 385.07108(77), 417.06088(7), 443.04037(36), 537.26947(10)	2	31
**73**	**Physcion**	7.43	C16H11O5–	283.06120	283.06104	0.56	165.01944(3), 239.07121(2), 283.02463(74), **283.06180**(100)	2	41
**74**	**Emodin**	8.71	C15H9O5–	269.04555	269.04537	0.65	225.0558(3), **269.04544**(100)	1	31
Acylphloroglucinols									
**75**	**Selancin E**	6.98	C21H27O5–	359.18640	359.18555	2.35	180.07918(12), 194.05833(41), 195.06612(81), 208.07396(63), 341.17508(34), **359.18573**(100)	2	42
**76**	**Madeleinol B**	8.34	C25H35O5–	415.24900	415.24833	1.62	180.07924(6), 194.05847(7), 208.07399(45), 275.12866(9), **415.24857**(100)	2	42
**77**	**Empetriferdinan A**	8.45	C20H27O4–	331.19148	331.19132	0.49	151.00366(10), 166.06348(35), **194.05830**(100), 331.19147(93)	2	43
**78**	**Hypermongone der. 1**	9.49	C31H47O7–	531.33273	531.33202	1.33	461.29141(2), **531.33203**(100)	2	44
**79**	**Hyperbeanol O**	10.14	C28H33O5–	449.23335	449.23300	0.78	277.14441(14), 381.24280(4), **449.23282**(100)	2	45
**80**	**Colupulone**	10.35	C25H35O4–	399.25408	399.25344	1.61	180.07913(9), 194.0584(3), 208.07397(48), **399.25348**(100)	2	46
**81**	**Tomoeone H**	10.3	C31H45O7–	529.31708	529.3164	1.12	245.11746(4), 383.22400(5), 441.26410(28), 459.27484(16), 497.29071(5),	2	45

		9			9		**529.31604**(100)		
**82**	**Hypermongone der. 2**	10.50	$C_{31}H_{45}O_5^-$	497.32725	497.32681	0.88	207.06619(7), 233.08186(3), 276.13663(4), 277.14407(3), 427.28452(3), **497.32684**(100)	2	[44]
**83**	**Adlupulone**	10.82	$C_{26}H_{37}O_4^-$	413.26973	413.26913	1.46	194.09473(9), 208.07401(10), 222.08963(46), **413.27029**(100)	2	[46]
**84**	**Hypermongone der. 3**	11.70	$C_{31}H_{45}O_5^-$	497.32725	497.32657	1.35	222.08958(7), 329.17566(18), 383.29492(5), **497.32669**(100)	22	[44]
**85**	**Bellumone L**	12.08	$C_{32}H_{47}O_4^-$	495.34798	495.34695	2.08	277.14423(14), 451.32013(12), **495.31055**(100)		[47]
Other metabolites									
**86**	**Disaccharide**	0.47	$C_{12}H_{21}O_{11}^-$	341.10894	341.10802	2.69	59.01379(65), 71.01382(38), **89.02437**(100), 101.02438(31), 113.02443(24), 119.03496(35)	2	[18]
**87**	**Quinic acid**	0.48	$C_7H_{11}O_6^-$	191.05611	191.05594	0.89	85.02945(9), 111.00887(2), 127.04002(3), 173.04555(2), **191.05594**(100)	1	[14]
**88**	**Hexose**	0.49	$C_6H_{11}O_6^-$	179.05610	179.05606	0.20	**59.01377**(100), 71.01380(43), 89.02434(39), 101.02433(6), 113.02439(7)	2	[18]
**89**	**Malic acid**	0.51	$C_4H_5O_5^-$	133.01420	133.01420	0.02	71.01384(37), 89.02440(7), **115.00371**(100), 133.01428(45)	2	[18]
**90**	**Fumaric acid**	0.52	$C_4H_3O_4^-$	115.00370	115.00367	0.27	**71.01380**(100), 115.00366(20)	2	[18]
**91**	**Trihydroxybenzophenone hexuronide**	4.55	$C_{19}H_{17}O_{10}^-$	405.08272	405.08188	2.08	85.02943(4), 113.02435(5), 151.00363(5), 185.06071(3), **229.05048**(100)	2	NA
**92**	**Verbascoside**	5.37	$C_{29}H_{35}O_{15}^-$	623.19814	623.19738	1.23	113.02443(11), 135.04512(8), **161.02441**(100), 179.03505(4), 461.16599(14), 623.19238(3)	2	[48]
**93**	**Trihydroxyoctadecadienoic acid**	7.01	$C_{18}H_{31}O_5^-$	327.21770	327.21751	0.58	221.08189(27), 239.09241(10), 309.20651(10), **327.21756**(100)	2	[21]
**94**	**Fusidic acid**	9.63	$C_{31}H_{47}O_6^-$	515.33781	515.33731	0.98	445.29733(3), **515.33789**(100)	2	[18]
**95**	**Hyperfirin**	11.57	$C_{30}H_{43}O_4^-$	467.31668	467.31618	1.09	301.216490(4), 399.25323(10), 411.29025(5), 423.32635(10), **467.31613**(100)	2	[31]
**96**	**Adhyperfirin**	11.8	$C_{31}H_{45}O_4^-$	481.33233	481.3318	1.10	233.08125(4), 245.15486(3), 276.13641(4), 411.29129(3), 437.34195(12),	2	[31]

		4			1		**481.33194**(100)		
**97**	**Adhyperforin**	12.87	C36H53O4–	549.39493	549.39416	1.41	**549.37915**(100)	2	[31]
**98**	**Hypericin**	13.33	C30H15O8–	503.07724	503.07690	0.69	**503.07651**(100)	2	[31]
**99**	**Pseudohypericin**	14.38	C30H15O9–	519.07216	519.07175	0.79	**519.07153**(100)	2	[31]

Confidence levels to reported metabolite were proposed according to Schymanski algoritm [49]: level 1 – confirmed with authentic reference standards (matching exact mass, MS/MS fragmentation, and retention time); level 2 – probable structure based on spectral library matching and literature data (accurate mass and MS/MS fragmentation).

Table S3. Peak areas of tentatively annotated compounds; Values were obtained from full scan MS spectra.

Compound name	*Hypericum ekerii* (Acetone water)	*Hypericum ekerii* (Acetone)	*Hypericum ekerii* (Ethyl acetate)	*Hypericum ekeri* (water)
**Hydroxybenzoic acid isomer**	112931645	240887449	67039365	90040626
**Gallic acid hexoside**	209697874	112431246	7410899	224361279
**Hydroxybenzoic acid hexoside**	47582626	105670240	31546889	34474473
**Gallic acid**	22084876	28703228	10488204	16497238
**Vanillic acid hexoside**	76894141	142021439	32822682	73685559
**Dihydroxybenzoic acid hexoside**	634108995	252943261	30134209	489590394
**3,4-Dihydroxybenzoic acid (Protocatechuic acid)**	239018372	471828084	106669649	216090797
**Ellagic acid**	29585204	573744	0	5766715
**Vanillic acid**	0	0	5073119	0
**p-Hydroxybenzoic acid**	113675935	0	49034072	83566052
**Caffeoylquinic acid hexoside**	35345202	6357569	582026	8999466
**Caffeic acid hexoside**	30452028	28386031	3964332	23465221
**5-O-Caffeoylquinic acid**	3743592567	1461146982	126328223	2854339215
**5-O-Coumaroylquinic acid**	1055987878	205772348	6744461	723740182
**p-Coumaric acid hexoside**	110018040	38009272	0	42492345
**Caffeic acid**	22846992	0	0	19063184
**p-Coumaric acid**	5347778	0	4034245	0
**Caffeoylshikimic acid**	96730759	57215686	40100066	11609543
**Procyanidin**	27255625	0	0	11280848
**(Epi)gallocatechin**	30871611	12697834	907050	25242338
**B type procyanidin dimer 1**	261302302	97909759	1319844	228239974
**(Epi)catechin**	1523759732	160811779	0	1076557242
**B type procyanidin dimer 2**	41870550	74288506	438903	0
**Apigenin 6,8-di-O-hexoside**	42564278	0	0	7005886
**Luteolin 8-C-hexoside-6-C-pentoside**	14344642	7742324	0	13024371
**Luteolin 8-C-glucoside**	459733842	374859435	24234108	256647266
**Chrysin 6,8-di-C-hexoside**	39783173	6938723	0	19904579
**Apigenin C-hexoside-C-pentoside (Iso)schaftoside**	9515219	5635603	0	7880581
**Apigenin 8-C-glucoside**	89810293	63198631	10317650	56583077
**Luteolin 8-C-(6"-malonyl)-hexoside**	20897048	69600460	5418010	29721328
**Myricetin 3-O-hexoside**	2309274840	1270374749	130494102	1360129109
**Myricetin 3-O-(6"-rhamnosyl)-hexoside**	185472087	65593013	2269975	108417253
**Quercetin 3-O-rhamnoside-7-O-hexuronide**	10486881	0	0	4219134
**Myricetin 3-O-hexuronide**	2124977071	366128761	17857542	770986699
**Quercetin 3-O-rhamnoside-7-O-hexoside**	35468469	36794413	756319	25145410
**Quercetin 3,7-di-O-hexoside**	15535213	0	0	0
**Quercetin 3-O-rhamoside-7-O-pentoside**	15118925	6828837	0	7292994
**Myricetin 3-O-pentoside**	77652477	31640830	2893164	36440007
**Quercetin 3-O-(2"-hexosyl)-hexuronide**	320202099	0	180888	0
**Quercetin 3-O-(6"-rhamnosyl)-glucoside**	1316877994	1559649533	127417708	1057985015

**Quercetin 3-O-glucoside**	1497005622	3646695501	740069838	1635922081
**Quercetin 3-O-hexuronide**	5309376150	5487608534	385597041	3525769078
**Quercetin 3-O-(6''-acetyl)-hexoside**	208976569	229881856	13818859	140099595
**Kaempferol 3-O-(6"-rhamnosyl)-hexoside**	277981394	280621484	37580398	193330487
**Kaempferol 3-O-glucoside**	195375059	205376513	48772596	135500497
**Quercetin 3-O-pentoside**	327705886	317603132	53753377	191103651
**Quercetin 3-O-rhamnoside**	633163764	638062415	94750752	429001533
**Kaempferol 3-O-hexuronide**	1074841940	846771716	60729823	628287583
**Kaempferol 3-O-pentoside**	27507910	23030759	4825111	14712695
**Eriodictyol 7-O-hexoside**	15466222	9431227	1709495	4442997
**Kaempferol 3-O-(6"-malonyl)-hexoside**	40871513	40794884	5022882	27520631
**Kaempferol 3-O-(6''-acetyl)-hexoside**	50408573	51289697	5917144	34425041
**Myricetin 3-O-(6''-p-coumaroyl)-hexoside**	22840970	9131389	0	8754088
**Quercetin 3-O-(2''-acetyl)-hexuronide**	1026248059	559832773	27787082	482754092
**Kaempferol 3-O-rhamnoside**	337188216	373929952	60177015	181702841
**Quercetin 3-O-(2''-p-coumaroyl)-hexoside**	112341769	82971670	11754994	41592958
**Myricetin**	596599877	75971439	39361239	104702687
**Luteolin**	143061615	158986428	114181761	30674102
**Quercetin**	1341394553	512731934	399146794	437474855
**Apigenin**	32162899	26948874	18549589	4833125
**Quercetin 3,7-dimethyl ether**	20988289	31737515	47349973	3754270
**Kaempferol**	209378115	149105171	124986204	48514919
**Chrysoeriol**	49691154	64545638	51928643	8639492
**Mangiferin**	27081564	31220716	0	0
**Dihydroxydimethoxyxanthone 1**	47527580	144532154	160871003	9323950
**Hydroxydimethoxyxanthone**	59065425	187774310	202060995	10091772
**Trihydroxydimethoxyxanthone**	189032767	273113170	298355804	27698857
**Dihydroxydimethoxyxanthone 2**	19788474	39460776	43730988	3697241
**Dihydroxytrimethoxyxanthone**	27097681	68419681	77521128	1989702
**5,ω-Dihydroxyemodin**	0	16120426	0	0
**Skyrin hexoside**	8913319	7107650	0	2735673
**Skyrin**	926127530	3380086839	2914219264	81555069
**Physcion**	9500297	17963231	16613677	1428708
**Emodin**	17110769	52545470	83485600	2316401
**Selancin E**	59407577	2035715294	918665828	0
**Madeleinol B**	490239549	4713374178	4623838181	84095026
**Empetriferdinan A**	388584097	233316465	5489648565	608511664
**Hypermongone der. 1**	344472714	5809173831	4025056550	66969600
**Hyperbeanol O**	160101137	697357435	3204349092	64304485
**Colupulone**	1508788453	760449453	26516700058	553474133
**Tomoeone H**	388525293	6230529333	5815768603	51597305
**Hypermongone der. 2**	494128174	0	10349325297	61528058
**Adlupulone**	157743586	74710106	2566185907	57663321
**Hypermongone der. 3**	1502747560	4812055771	18481257904	123928637
**Bellumone L**	313129355	44920684	11244312013	210718745
**Disaccharide**	73007834	1296017684	154867099	304540909

**Quinic acid**	9155030813	5078660479	1034864739	7821353570
**Hexose**	663305789	364377929	96431662	376807991
**Malic acid**	1056593487	867202389	9014890	911358108
**Fumaric acid**	251291949	226592678	3879507	226341981
**Trihydroxybenzophenone hexuronide**	2547878172	2410551837	73231423	1622939585
**Verbascoside**	6998649	19271857	9945510	8535947
**Trihydroxyoctadecadienoic acid**	581347688	217815201	236004711	549969542
**Fusidic acid**	189959416	0	4528174394	155704326
**Hyperfirin**	24142802	0	1461267674	21711383
**Adhyperfirin**	3561089674	723654889	92211996507	2216898759
**Adhyperforin**	506123	0	29853037	341388
**Hypericin**	67021994	0	0	0
**Pseudohypericin**	230210118	28043536	0	98554129

Table S4. Scaffold split performance of external ChEMBL-based QSAR models used in the prioritization.

Module	Target	ChEMBL ID	Molecule-level rows	Active / inactive_or_weak	ROC-AUC	PR-AUC	F1-score	MCC	Balanced accuracy	Interpretation
**hCA**	hCA I	CHEMBL261	6817	4362 / 2455	0.8501	0.9121	0.8520	0.5430	0.7669	Good external QSAR layer
**hCA**	hCA II	CHEMBL205	7695	6620 / 1075	0.9120	0.9803	0.9365	0.5355	0.7413	Good external QSAR layer
**Cholinesterase**	AChE	CHEMBL220	5175	2470 / 2705	0.8739	0.8524	0.7341	0.5686	0.7744	Good external QSAR layer
**Cholinesterase**	BChE	CHEMBL1914	3292	1498 / 1794	0.8902	0.9059	0.8110	0.6307	0.8150	Good external QSAR layer
**Tyrosinase**	Mushroom tyrosinase	CHEMBL3318	1033	219 / 814	0.8189	0.7376	0.6163	0.5401	0.7259	Usable but active-class recall caution

Table S5. Integrated candidate evidence summary for prioritized *Hypericum ekerii* metabolites.

Candidate	Evidence tier	Manuscript-use label	Main evidence support	Dominant extract	Suggested interpretation
**Myricetin**	Tier 1A	Cross-layer priority candidate	Reliable AChE-selective QSAR support; exploratory hCA support; strong integrated antioxidant support	Hydroacetone	Highest-priority hypothesis-generating candidate supported by both target-specific QSAR and antioxidant structure–pattern layers.
**p-Coumaric acid**	Tier 1B	Target-specific priority candidate	Reliable hCA II QSAR support; moderate integrated antioxidant support	Hydroacetone	hCA II-focused priority candidate with moderate antioxidant support.
**Ellagic acid**	Tier 2A	Antioxidant-priority candidate	Strong integrated antioxidant support; no reliable target-specific QSAR support in tested enzyme modules	Hydroacetone	Best interpreted as an antioxidant-priority metabolite rather than an enzyme-target candidate.
**Quercetin**	Tier 2A	Antioxidant-priority candidate	Strong integrated antioxidant support; no reliable target-specific QSAR support in tested enzyme modules	Hydroacetone	Strong antioxidant-priority candidate; target-specific enzyme support was not observed.
**Caffeic acid**	Tier 2A	Antioxidant-priority candidate	Strong integrated antioxidant support; no reliable target-specific QSAR support in tested enzyme modules	Hydroacetone	Strong antioxidant-priority candidate consistent with phenolic/catechol antioxidant chemistry.
**Apigenin**	Tier 2B	Exploratory target/antioxidant candidate	Exploratory hCA support; moderate integrated antioxidant support	Hydroacetone	Preliminary candidate; target-specific evidence should be interpreted cautiously.
**Empetriferdinan A**	Tier 3A	Exploratory target-specific candidate	Exploratory hCA support; low integrated antioxidant support	EtOAc	Preliminary hCA-related candidate requiring further validation.
**Fusidic acid**	Tier 3A	Exploratory target-specific candidate	Exploratory hCA support; low integrated antioxidant support	EtOAc	AD-limited exploratory hCA candidate; should not be overinterpreted.
**Adlupulone**	Tier 3A	Exploratory target-specific candidate	Exploratory hCA support; low integrated antioxidant support	EtOAc	Preliminary hCA-related candidate requiring further validation.
**Adhyperfirin**	Tier 3A	Exploratory target-specific candidate	Exploratory hCA support; low integrated antioxidant support	EtOAc	Preliminary hCA-related candidate; interpretation should remain cautious.
**Hyperfirin**	Tier 3A	Exploratory target-specific candidate	Exploratory hCA support; low integrated antioxidant support	EtOAc	Preliminary hCA-related candidate; not supported as an antioxidant-priority compound.
**Adhyperforin**	Tier 3A	Exploratory target-specific candidate	Exploratory hCA support; low integrated antioxidant support	EtOAc	Preliminary hCA-related candidate requiring experimental confirmation.
**Vanillic acid**	Tier 4	Lower-priority candidate	No reliable target-specific QSAR support; low integrated antioxidant support	EtOAc	Lower-priority candidate under the current integrated framework.
**Colupulone**	Tier 4	Lower-priority candidate	No reliable target-specific QSAR support; low integrated antioxidant support	EtOAc	Lower-priority candidate under the current evidence layers.

Table S6. The docking score (kcal/mol) and interacting residues of the enzyme.

Compound	Target	PDB ID	Binding energy	RMSD	Type and Binding site
5-O-caffeoylquinic acid	AChE	7E3H	-9.0	0.0	Hbond: TYR:133;TYR:337<br>Hydrophobic: TRP:86;TRP:86;TRP:86;PHE:297<br>Salt bridge: HIS:447
Adhyperfirin	AChE	7E3H	-8.6	1.0	Hbond: TYR:124;TYR:337;TYR:337<br>Hydrophobic: ASP:74;TRP:86;TRP:86;TRP:86;TYR:124;TYR:124;ALA:204;TRP:236;TRP:236;PHE:295;PHE:297;PHE:297;TYR:337;PHE:338;PHE:338;PHE:338;TYR:341;TYR:341
Adhyperforin	AChE	7E3H	-7.0	3.5	Hbond: TYR:124<br>Hydrophobic: ASP:74;TRP:86;TRP:86;TRP:86;TRP:86;TRP:86;TYR:124;TYR:124;LEU:130;TYR:133;PHE:295;PHE:297;PHE:338;PHE:338;PHE:338;TYR:341;TYR:341;TYR:341;ILE:451
Adlupulone	AChE	7E3H	-9.2	0.6	Hbond: TYR:341<br>Hydrophobic: TYR:72;ASP:74;TRP:86;TRP:86;TRP:86;TYR:124;TYR:124;PHE:295;PHE:297;TYR:337;PHE:338;PHE:338;PHE:338;TYR:341;TYR:341
Epicatechin	AChE	7E3H	-9.3	1.1	Hbond: GLY:120;GLY:120;TYR:124;TYR:124;TYR:133;TYR:133;TYR:337;TYR:337<br>Hydrophobic: TRP:86;TYR:337;PHE:338;PHE:338;TYR:341
Hyperfirin	AChE	7E3H	-7.9	4.9	Hbond: TYR:337<br>Hydrophobic: ASP:74;TRP:86;TRP:86;TRP:86;TYR:124;TYR:133;TRP:286;TYR:337;PHE:338;PHE:338;TYR:341;TYR:341;ILE:451
Hypericin	AChE	7E3H	-2.0	1.0	Hbond: GLN:71;ASP:74;TRP:86;TYR:124;GLY:126;ALA:127;TYR:133<br>Hydrophobic: ASP:74;TRP:86;LEU:130;TYR:337;PHE:338;PHE:338;HIS:447<br>Pi-stacking: TYR:124;TYR:124

Myricetin-3-O-hexoside	AChE	7E3H	-8.1	0.6	Hbond: GLY:120;GLY:120;GLY:121;TYR:124;GLY:126;TYR:133;GLU:202;GLU:202;PHE:295<br>Hydrophobic: PHE:297;TYR:337;PHE:338;TYR:341
Myricetin-3-O-hexuronide	AChE	7E3H	-8.3	0.6	Hbond: GLY:121;TYR:124;TYR:124;SER:203;PHE:295;ARG:296;HIS:447<br>Hydrophobic: PHE:297;TYR:337;PHE:338<br>Pi-cation: HIS:447
Pseudohypericin	AChE	7E3H	-2.2	0.3	Hbond: ASP:74;TRP:86;GLY:126;ALA:127;TYR:133;TYR:133<br>Hydrophobic: TRP:86;LEU:130;TYR:337;PHE:338<br>Pi-stacking: TYR:124;TYR:124
Quercetin-3-O-hexoside	AChE	7E3H	-8.4	1.0	Hbond: ASP:74;TRP:86;GLY:120;GLY:121;SER:125;PHE:295;ARG:296;TYR:337<br>Hydrophobic: TRP:86;PHE:297;TYR:337;PHE:338<br>Pi-cation: HIS:447
Tomoeone H	AChE	7E3H	-2.5	0.1	Hbond: TYR:72;TYR:124;SER:125;SER:125<br>Hydrophobic: ASP:74;TRP:86;TRP:86;TRP:86;TRP:236;PHE:295;PHE:297;PHE:297;TYR:337;TYR:337;TYR:337;PHE:338;PHE:338;TYR:341;TYR:341
Apigenin	AChE	7E3H	-9.3	0.6	Hbond: TRP:86;TYR:133;GLU:202<br>Hydrophobic: TRP:86;TRP:86;PHE:297;PHE:338<br>Pi-stacking: TYR:341
Myricetin	AChE	7E3H	-9.9	0.9	Hbond: GLN:71;ASP:74;ASP:74;GLY:120;TYR:133;TYR:133;TYR:337;HIS:447<br>Hydrophobic: TRP:86<br>Pi-stacking: TRP:86;TRP:86
Pcoumaric acid	AChE	7E3H	-7.1	1.0	Hbond: GLY:120;TYR:124;TYR:124;TYR:133;TYR:133;TYR:337<br>Hydrophobic: TRP:86;TRP:86<br>Pi-stacking: TRP:86
5-O-caffeoylquinic acid	Amylase	2QV4	-8.2	8.9	Hbond: GLN:63;GLY:104;ARG:195;ALA:198;GLU:233;GLU:233;HIS:299<br>Hydrophobic: TRP:59;TRP:59;TYR:62;TYR:62

Adhyperfirin	Amylase	2QV4	-7.8	0.7	Hbond: HIS:305<br>Hydrophobic: TRP:59;TRP:59;TRP:59;TRP:59;TYR:62;TYR:62;THR:163;LEU:165;ALA:198
Adhyperforin	Amylase	2QV4	-8.0	1.0	Hydrophobic: TRP:58;TRP:58;TRP:59;TRP:59;TYR:62;TYR:62;TYR:62;LEU:162;LEU:162;LEU:165;LYS:200;LYS:200;ILE:235;ILE:235;ILE:235
Adlupulone	Amylase	2QV4	-7.7	2.3	Hbond: TYR:62;GLN:63<br>Hydrophobic: TRP:59;TRP:59;TRP:59;TRP:59;TRP:59;TRP:59;TYR:62;TYR:62;TYR:62;LEU:165;LEU:165
Epicatechin	Amylase	2QV4	-8.5	0.8	Hbond: ARG:195;ASP:197;HIS:299;ASP:300<br>Hydrophobic: TRP:58;TRP:59;TYR:62<br>Pi-stacking: TRP:59;TRP:59;TYR:62
Hyperfirin	Amylase	2QV4	-8.4	0.7	Hbond: ASP:300<br>Hydrophobic: TRP:58;TRP:59;TRP:59;TRP:59;LEU:162;LEU:162;THR:163;LEU:165;LEU:165;ALA:198;ILE:235
Hypericin	Amylase	2QV4	-10.8	0.7	Hbond: HIS:101;ASP:197;GLU:233<br>Hydrophobic: TRP:59;LEU:165;LEU:165
Myricetin-3-O-hexoside	Amylase	2QV4	-8.9	0.5	Hbond: ARG:195;ASP:197;LYS:200;HIS:201;GLU:233;HIS:299;ASP:300;HIS:305
Myricetin-3-O-hexuronide	Amylase	2QV4	-8.2	0.4	Hbond: TRP:59;GLN:63;ARG:195;ALA:198;GLU:233;HIS:299;ASP:300;HIS:305<br>Hydrophobic: THR:163;LEU:165<br>Salt bridge: HIS:201
Pseudohypericin	Amylase	2QV4	-10.4	0.8	Hbond: HIS:101;THR:163;THR:163;ASP:197;GLU:233<br>Hydrophobic: TRP:59;LEU:165;LEU:165
Quercetin-3-O-hexoside	Amylase	2QV4	-8.4	1.1	Hbond: GLN:63;THR:163;ARG:195;ARG:195;ASP:197;HIS:201;GLU:233;HIS:299;HIS:305;HIS:305;HIS:305

					Hydrophobic: TRP:58;TYR:62;LEU:165
Tomoeone H	Amylase	2QV4	-7.7	0.3	Hbond: GLN:63<br>Hydrophobic: ILE:51;TRP:58;TRP:59;TRP:59;TYR:62;VAL:107;LEU:162
5-O-caffeoylquinic acid	BChE	6EQP	-8.8	1.0	Hbond: GLY:115;TYR:128;TYR:128;LEU:286<br>Hydrophobic: TRP:82;TRP:82<br>Pi-stacking: PHE:329
Apigenin	Amylase	2qv4	-9.0	0.6	Hbond: GLN:63;ARG:195;ARG:195;HIS:299<br>Hydrophobic: TRP:58;TRP:59;TYR:62<br>Pi-stacking: TRP:59;TRP:59;TYR:62
Myricetin	Amylase	2qv4	-9.3	0.2	Hbond: GLN:63;ARG:195;GLU:233;HIS:299;ASP:300<br>Hydrophobic: TRP:58;TYR:62;TYR:62<br>Pi-stacking: TRP:59;TRP:59
Pcoumaric acid	Amylase	2qv4	-6.2	0.3	Hbond: GLN:63;ARG:195;ASP:197;HIS:299<br>Hydrophobic: TRP:58;TYR:62;LEU:165<br>Pi-stacking: TYR:62
Adhyperfirin	BChE	6EQP	-9.8	1.1	Hbond: THR:120;SER:198<br>Hydrophobic:<br>TRP:82;TRP:82;TRP:82;TRP:231;LEU:286;VAL:288;PHE:329;PHE:329;PHE:329;TYR:332;PHE:398;TRP:430
Adhyperforin	BChE	6EQP	-9.9	1.0	Hbond: ASP:70<br>Hydrophobic:<br>ASP:70;TRP:82;TRP:82;TRP:231;TRP:231;LEU:286;ALA:328;PHE:329;TYR:332;PHE:398;TYR:440
Adlupulone	BChE	6EQP	-9.0	2.5	Hbond: GLY:116;GLY:117;SER:198<br>Hydrophobic:<br>ASP:70;TRP:82;TRP:82;TRP:82;TRP:231;LEU:286;VAL:288;ALA:328;PHE:329;TYR:332;PHE:398;TRP:430

Epicatechin	BChE	6EQP	-8.1	0.2	Hbond: TRP:82;GLY:117;SER:198;SER:198;TRP:430;TYR:440<br>Hydrophobic: PHE:329;PHE:329;TYR:332;TRP:430
Hyperfirin	BChE	6EQP	-8.9	0.0	Hbond: GLY:116<br>Hydrophobic:<br>ASP:70;TRP:82;TRP:82;LEU:286;ALA:328;PHE:329;PHE:329;TYR:332;TYR:440
Hypericin	BChE	6EQP	-4.6	0.5	Hbond:<br>GLN:67;ASP:70;GLY:115;GLY:121;GLY:121;TYR:128;TYR:128;GLU:197;HIS:438;HIS:438<br>Hydrophobic: ASP:70;TRP:82;LEU:125;TYR:332;ILE:442<br>Pi-stacking: TRP:82;TRP:82;TRP:82;TRP:82;TRP:82
Myricetin-3-O-hexoside	BChE	6EQP	-10.5	1.1	Hbond: ASP:70;GLY:115;TYR:128;GLU:197;SER:198;SER:198;SER:287;TYR:332<br>Pi-stacking: PHE:329;PHE:329;TYR:332
Myricetin-3-O-hexuronide	BChE	6EQP	-10.8	0.9	Hbond:<br>ASP:70;SER:79;TRP:82;GLY:115;GLY:115;GLY:115;GLY:117;TYR:128;SER:198;SER:287;<br>HIS:438<br>Pi-stacking: PHE:329
Pseudohypericin	BChE	6EQP	-4.7	1.0	Hbond: ASP:70;ASP:70;GLY:115;GLY:121;GLY:121;TYR:128;TYR:128;HIS:438<br>Hydrophobic: ASP:70;TRP:82;LEU:125;ILE:442<br>Pi-stacking: TRP:82;TRP:82;TRP:82;TRP:82;TRP:82
Quercetin-3-O-hexoside	BChE	6EQP	-10.6	1.0	Hbond:<br>ASP:70;ASP:70;ASP:70;GLY:115;GLY:115;GLY:117;TYR:128;GLU:197;GLU:197;SER:198<br>;LEU:286<br>Hydrophobic: TRP:82<br>Pi-stacking: PHE:329;PHE:329
Tomoeone H	BChE	6EQP	-9.7	0.6	Hbond: GLY:117;PRO:285<br>Hydrophobic: TRP:82;ALA:328;TYR:332;PHE:398;TYR:440
Apigenin	BChE	6EQP	-9.3	0.2	Hbond: THR:120;GLU:197<br>Hydrophobic: TRP:82;TRP:82;TYR:332;TRP:430<br>Pi-stacking: TRP:82;TRP:82

Myricetin	BChE	6EQP	-8.8	1.0	Hbond: GLY:115;THR:120;TYR:128;GLU:197;ALA:328<br>Hydrophobic: TRP:82;PHE:329<br>Pi-stacking: TRP:82;TRP:82
Pcoumaric acid	BChE	6EQP	-6.3	0.7	Hbond: ASP:70;GLY:115;GLY:115<br>Hydrophobic: TRP:82;TRP:82<br>Pi-stacking: TRP:82
5-O-caffeoylquinic acid	Glucosidase	7KBJ	-8.3	0.5	Hbond: ASP:251;GLU:287;GLU:287;MET:289;SER:290;ARG:334;ARG:334;ALA:345;ARG:352;ARG:352<br>Hydrophobic: LEU:252;TYR:291;TRP:331<br>Salt bridge: LYS:283;ARG:352
Adhyperfirin	Glucosidase	7KBJ	-5.2	4.7	Hbond: LEU:252;GLY:253;GLY:254<br>Hydrophobic: MET:289;TYR:291;TYR:291;TRP:331;LEU:344
Adlupulone	Glucosidase	7KBJ	-6.6	0.9	Hbond: LEU:252;GLY:253;TYR:291<br>Hydrophobic: LEU:252;LEU:252;ILE:257;LEU:344;PHE:350;PHE:350
Epicatechin	Glucosidase	7KBJ	-8.0	0.7	Hbond: GLU:248;LYS:283;GLU:287;TYR:291;TYR:291;ARG:334;ARG:352;ARG:352<br>Hydrophobic: LEU:252
Hyperfirin	Glucosidase	7KBJ	-3.9	0.6	Hbond: GLY:253;GLY:254;GLU:287;SER:290;ARG:352;ARG:352<br>Hydrophobic: PHE:237;LEU:252;LEU:252;GLU:270;TYR:291;ARG:352
Myricetin-3-O-hexoside	Glucosidase	7KBJ	-6.7	6.9	Hbond: GLY:254;GLY:264;GLY:264;TYR:348;PHE:350;ARG:352;ARG:352;ARG:352;ARG:352<br>Hydrophobic: TYR:291;TYR:291;TRP:331<br>Salt bridge: ARG:352
Myricetin-3-O-hexuronide	Glucosidase	7KBJ	-7.7	1.1	Hbond: GLU:248;LEU:252;GLY:253;GLY:254;ASN:265;GLU:287;ARG:334;ARG:334;PHE:350;ARG:352<br>Hydrophobic: TRP:331<br>Pi-stacking: TYR:291

					Salt bridge: ARG:352;ARG:352
Quercetin-3-O-hexoside	Glucosidase	7KBJ	-8.3	0.9	Hbond: LEU:252;GLY:253;GLY:254;ASN:265;TYR:291;ARG:334;ARG:334;PHE:350;ARG:352;ARG:352<br>Hydrophobic: TRP:331<br>Pi-stacking: TYR:291<br>Salt bridge: ARG:352
Tomoeone H	Glucosidase	7KBJ	-5.3	6.1	Hbond: GLY:253;GLY:254;LYS:283;GLU:287;ARG:352;ARG:352<br>Hydrophobic: LEU:252;ILE:257;ILE:257;TYR:291;TYR:291;LEU:344;PHE:350;PHE:350
Apigenin	Glucosidase	7KBJ	-8.2	0.1	Hbond: ASN:265;ARG:334;LEU:344;ARG:352<br>Hydrophobic: ILE:257;TYR:291
Myricetin 5281672	Glucosidase	7KBJ	-7.7	2.9	Hbond: GLU:248;GLY:264;TYR:291;TYR:291;TYR:291;ARG:334;ARG:352<br>Pi-stacking: TYR:291
Pcoumaric acid	Glucosidase	7KBJ	-5.5	0.8	Hbond: PHE:94;GLY:220<br>Hydrophobic: PHE:32;ARG:210
5-O-caffeoylquinic acid	hCAI	3LXE	-7.5	0.4	Hbond: SER:65;PHE:66;PHE:66;GLN:92<br>Hydrophobic: VAL:62;PHE:91;LEU:198;HIS:200<br>Salt bridge: HIS:64;HIS:67;HIS:94;HIS:96;HIS:200
Adhyperfirin	hCAI	3LXE	-8.8	0.7	Hbond: HIS:64;GLN:92;HIS:200<br>Hydrophobic: TRP:5;VAL:62;VAL:62;VAL:62;HIS:67;PHE:91;HIS:94;HIS:119;LEU:131;LEU:141;VAL:143;VAL:143;LEU:198;LEU:198;HIS:200;VAL:207;TRP:209;TRP:209
Adhyperforin	hCAI	3LXE	-7.7	1.1	Hbond: HIS:64;GLN:92<br>Hydrophobic: TRP:5;TRP:5;HIS:67;HIS:94;LEU:131;ALA:135;LEU:198;LEU:198;LEU:198;HIS:200
Adlupulone	hCAI	3LXE	-7.6	1.7	Hbond: HIS:64;GLN:92;PRO:201<br>Hydrophobic: VAL:62;PHE:91;PHE:91;LEU:131;LEU:198;LEU:198;TRP:209

Epicatechin	hCAI	3LXE	-7.5	0.4	Hbond: TYR:7;TYR:7;ASP:8;ASN:11;HIS:64;LYS:170;GLN:242;HIS:243<br>Hydrophobic: HIS:64
Hyperfirin	hCAI	3LXE	-8.2	0.3	Hbond: GLN:92<br>Hydrophobic: TRP:5;VAL:62;PHE:91;ALA:121;ALA:135;VAL:143;LEU:198;LEU:198;TYR:204;VAL:207;TRP:209;TRP:209
Hypericin	hCAI	3LXE	-8.9	1.1	Hbond: TYR:7;HIS:64;HIS:94;HIS:96;HIS:119;THR:199<br>Hydrophobic: TRP:5;PHE:91;LEU:141;LEU:198;HIS:200;TRP:209
Myricetin-3-O-hexoside	hCAI	3LXE	-8.4	0.1	Hbond: TYR:7;HIS:64;SER:65;SER:65;SER:65;PHE:66;HIS:94;HIS:96;HIS:119;THR:199;THR:199<br>Hydrophobic: HIS:200<br>Pi-stacking: HIS:64;HIS:94;HIS:94<br>Salt bridge: HIS:94
Myricetin-3-O-hexuronide	hCAI	3LXE	-9.5	1.0	Hbond: HIS:64;SER:65;PHE:66;GLN:92;THR:199;THR:199;THR:199<br>Hydrophobic: TRP:5;HIS:200<br>Pi-stacking: HIS:64;HIS:94<br>Salt bridge: HIS:94;HIS:94;HIS:96;HIS:119
Pseudohypericin	hCAI	3LXE	-8.1	0.8	Hbond: TYR:7;HIS:64;HIS:94;HIS:96;GLU:106;HIS:119;THR:199<br>Hydrophobic: TRP:5;PHE:91;LEU:141;LEU:198;HIS:200
Quercetin-3-O-hexoside	hCAI	3LXE	-8.3	0.8	Hbond: HIS:64;GLN:92;HIS:96;THR:199;THR:199;THR:199;THR:199<br>Hydrophobic: ALA:135;HIS:200<br>Pi-stacking: HIS:94<br>Salt bridge: HIS:94
Tomoeone H	hCAI	3LXE	-8.0	0.6	Hbond: HIS:67;HIS:200<br>Hydrophobic: TRP:5;PHE:91;PHE:91;PHE:91;LEU:131;LEU:131;ALA:135;LEU:141;LEU:198;LEU:198;HIS:200;PRO:202;TYR:204
Apigenin	hCAI	3LXE	-7.9	0.6	Hbond: HIS:64;SER:65;HIS:94;HIS:96;THR:199;HIS:200

					Hydrophobic: PHE:91;LEU:131;LEU:198;LEU:198;HIS:200<br>Pi-stacking: HIS:94<br>Pi-cation: HIS:200
Myricetin	hCAI	3LXE	-7.5	5.5	Hbond: SER:65;HIS:96;THR:199;THR:199;HIS:200<br>Hydrophobic: LEU:198<br>Pi-stacking: HIS:94;HIS:94
Pcoumaric acid	hCAI	3LXE	-5.9	1.2	Hbond: GLN:242;HIS:243<br>Hydrophobic: TYR:7;HIS:64;MET:241<br>Salt bridge: LYS:170
5-O-caffeoylquinic acid	hCAII	4IWZ	-7.8	3.6	Hbond: ASN:62;HIS:64;ASN:67;GLN:92;HIS:94;HIS:96;HIS:119;THR:200<br>Hydrophobic: VAL:121;VAL:143;LEU:198;LEU:198<br>Salt bridge: HIS:94<br>Pi-cation: HIS:94
Adhyperfirin	hCAII	4IWZ	-8.7	0.9	Hbond: ASN:67;GLN:92<br>Hydrophobic: ASN:62;ALA:65;HIS:119;VAL:121;PHE:131;VAL:135;VAL:143;VAL:143;LEU:198;THR:200;PRO:202;LEU:204;VAL:207;TRP:209;TRP:209
Adhyperforin	hCAII	4IWZ	-8.4	0.1	Hbond: ASN:67<br>Hydrophobic: HIS:64;ALA:65;ILE:91;VAL:121;PHE:131;VAL:143;LEU:198;LEU:198;THR:200
Adlupulone	hCAII	4IWZ	-7.7	0.2	Hbond: GLN:92<br>Hydrophobic: HIS:64;ILE:91;VAL:121;PHE:131;VAL:135;VAL:143;LEU:198;LEU:198;THR:200
Epicatechin	hCAII	4IWZ	-7.8	1.1	Hbond: HIS:64;HIS:94;HIS:96;HIS:119;THR:200;PRO:201<br>Hydrophobic: VAL:121;LEU:198<br>Pi-stacking: HIS:94
Hyperfirin	hCAII	4IWZ	-7.6	0.3	Hbond: ASN:62;ASN:67;GLN:92;THR:200<br>Hydrophobic: GLN:92;VAL:121;PHE:131;PHE:131;LEU:198;THR:200

Hypericin	hCAII	4IWZ	-8.8	0.8	Hbond: TYR:7;GLY:8;LYS:170<br>Hydrophobic: LYS:170;PHE:231
Myricetin-3-O-hexoside	hCAII	4IWZ	-8.4	1.0	Hbond: ASN:62;HIS:64;GLN:92;HIS:119;THR:199;THR:200;THR:200<br>Hydrophobic: VAL:121;LEU:198;LEU:198<br>Pi-stacking: HIS:94;PHE:131;PHE:131
Myricetin-3-O-hexuronide	hCAII	4IWZ	-8.2	1.1	Hbond: TRP:5;HIS:64;HIS:94;HIS:96;THR:199;THR:199;THR:199;THR:200<br>Hydrophobic: GLN:92;LEU:198<br>Pi-stacking: PHE:131<br>Salt bridge: HIS:64
Pseudohypericin	hCAII	4IWZ	-8.9	0.9	Hbond: TYR:7;GLY:8;HIS:10;ASN:11;ASN:11;GLY:63;LYS:170<br>Hydrophobic: PHE:231
Quercetin-3-O-hexoside	hCAII	4IWZ	-8.7	0.4	Hbond: HIS:64;ASN:67;GLN:92;HIS:94;HIS:119;THR:199;THR:199;THR:200<br>Hydrophobic: VAL:121;LEU:198;LEU:198<br>Pi-stacking: HIS:94;PHE:131;PHE:131
Tomoeone H	hCAII	4IWZ	-8.3	1.0	Hbond: ASN:62;ASN:67;GLN:92;THR:200<br>Hydrophobic: TRP:5;HIS:64;PHE:131;PHE:131;VAL:135;THR:200;PRO:202
Apigenin	hCAII	4IWZ	-7.9	0.8	Hbond: ASN:62;HIS:64;GLN:92;HIS:94;HIS:96;HIS:119;THR:199<br>Hydrophobic: TRP:5;HIS:64;THR:200<br>Pi-cation: HIS:94
Myricetin	hCAII	4IWZ	-8.0	1.1	Hbond: ASN:62;ASN:62;HIS:64;HIS:94;HIS:96;HIS:119;THR:199;THR:199;PRO:201<br>Hydrophobic: THR:200<br>Pi-cation: HIS:94
Pcoumaric acid	hCAII	4IWZ	-5.9	1.1	Hydrophobic: TYR:7;PHE:231<br>Pi-stacking: PHE:231<br>Salt bridge: HIS:64;LYS:170
5-O-caffeoylquinic	Tyrosinase	6QXD	-6.0	0.5	Hbond: HIS:60;VAL:217<br>Hydrophobic: HIS:208;VAL:218<br>Pi-stacking: HIS:208

acid					Salt bridge: ARG:209
Adhyperfirin	Tyrosinase	6QXD	-4.4	0.8	Hbond: ARG:209<br>Hydrophobic: HIS:60;PHE:197;HIS:208
Adhyperforin	Tyrosinase	6QXD	-4.0	2.1	Hbond: VAL:218<br>Hydrophobic: HIS:60;PHE:197;HIS:208;VAL:218
Adlupulone	Tyrosinase	6QXD	-4.3	0.6	Hbond: VAL:218<br>Hydrophobic: PHE:197;HIS:208;VAL:218
Epicatechin	Tyrosinase	6QXD	-6.2	0.6	Hbond: ASN:205;ARG:209;ARG:209;MET:215<br>Pi-stacking: HIS:208<br>Pi-cation: ARG:209
Hyperfirin	Tyrosinase	6QXD	-4.9	0.2	Hbond: VAL:218<br>Hydrophobic: PHE:197;VAL:217;VAL:218;VAL:218;ALA:221
Hypericin	Tyrosinase	6QXD	-1.5	7.3	Hbond: ASN:205<br>Hydrophobic: HIS:60;PHE:197;ARG:209;VAL:218
Myricetin-3-O-hexoside	Tyrosinase	6QXD	-4.8	6.7	Hbond: HIS:60;GLU:195;HIS:208;ARG:209<br>Hydrophobic: VAL:218
Myricetin-3-O-hexuronide	Tyrosinase	6QXD	-5.0	0.9	Hbond: ASN:205;ASN:205;HIS:208<br>Pi-stacking: HIS:208
Pseudohypericin	Tyrosinase	6QXD	-1.6	0.4	Hbond: ASN:57;GLU:195;ASN:205;GLY:216<br>Hydrophobic: ARG:209;VAL:218
Quercetin-3-O-hexoside	Tyrosinase	6QXD	-5.3	0.5	Hbond: ASN:205;ARG:209;ARG:209;GLY:216<br>Hydrophobic: VAL:217;VAL:218
Tomoeone H	Tyrosinase	6QXD	-3.7	4.3	Hbond: ARG:209;ARG:209<br>Hydrophobic: HIS:208;VAL:217;VAL:218
Apigenin	Tyrosinase	6QXD	-6.0	0.5	Hbond: MET:61;GLU:195

					Hydrophobic: PHE:197;VAL:218
Myricetin	Tyrosinase	6QXD	-6.3	3.6	Hbond: ARG:209;VAL:217<br>Pi-stacking: HIS:208<br>Pi-cation: ARG:209
Pcoumaric acid	Tyrosinase	6QXD	-5.7	0.6	Hbond: ARG:209;VAL:217<br>Hydrophobic: PHE:197;ASN:205;VAL:218<br>Salt bridge: HIS:208

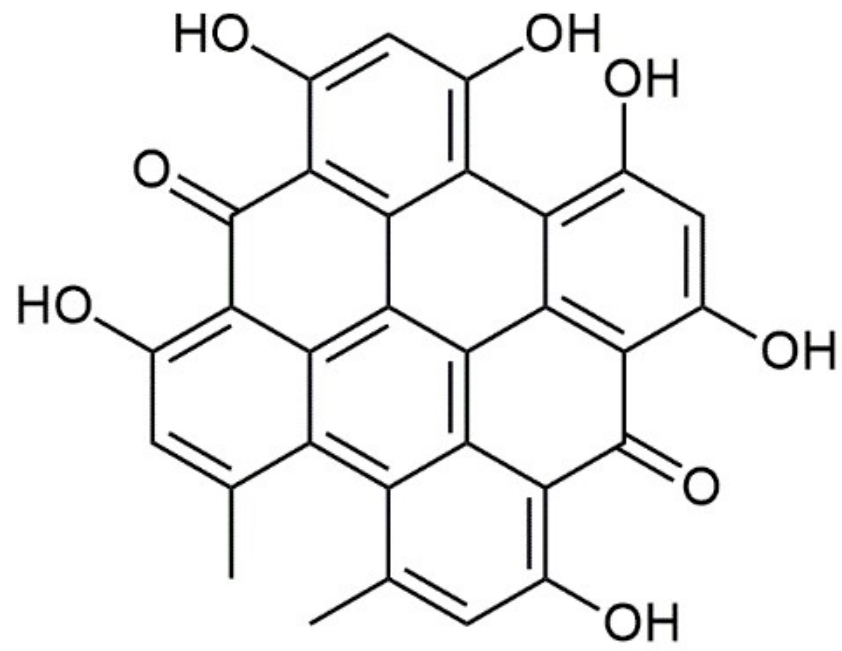

Hypericin
(naphthodianthrone)

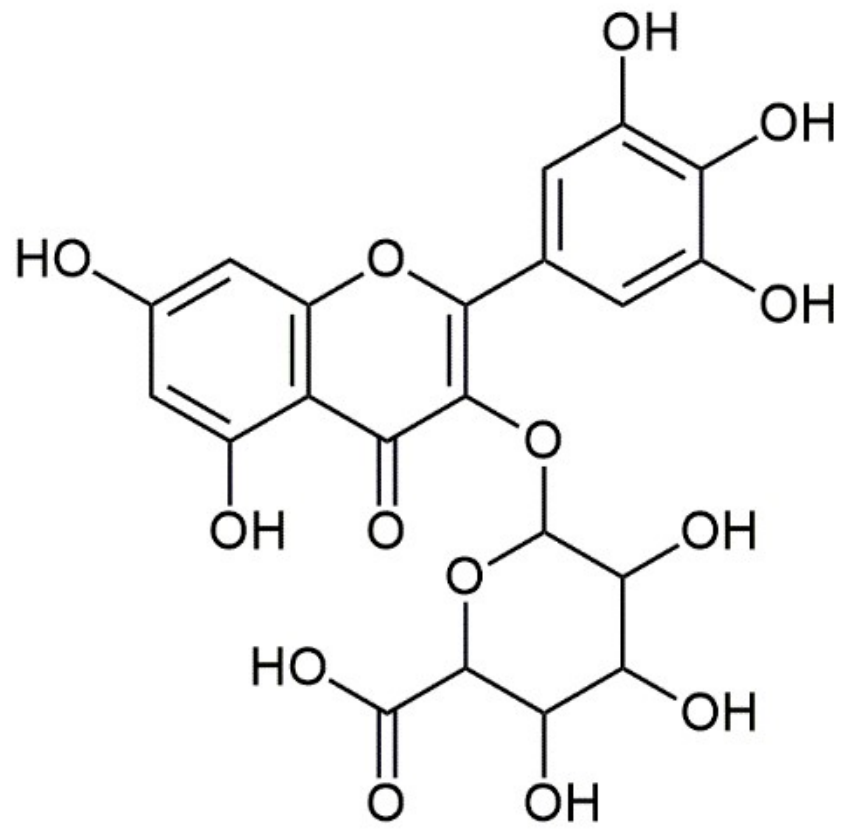

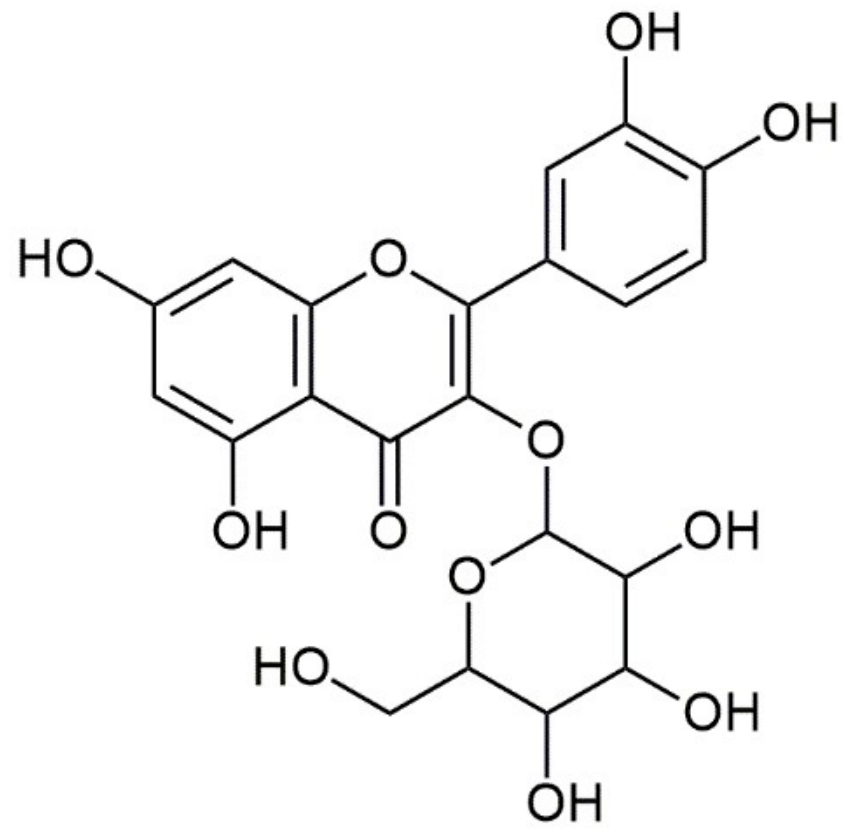

Myricetin 3-O-hexuronide

Quercetin 3-O-hexoside

Flavonoid glycosides

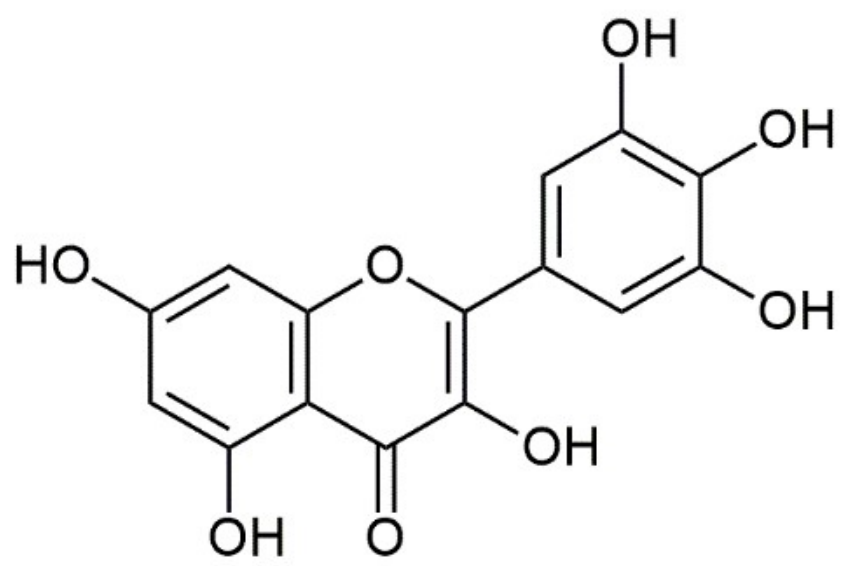

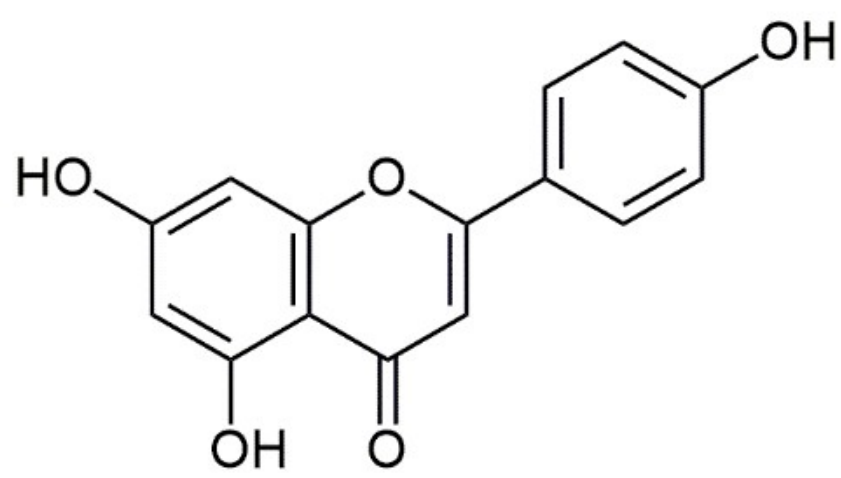

Myricetin

Apigenin

Flavonoid aglycones

Figure S1. Chemical structures of the key bioactive compounds identified in the plant extracts, categorized by their respective compound classes: naphthodianthrones (hypericin), flavonoid glycosides (myricetin-3-O-hexuronide, quercetin-3-O-hexoside), and flavonol - myricetin, and flavone apigenin).

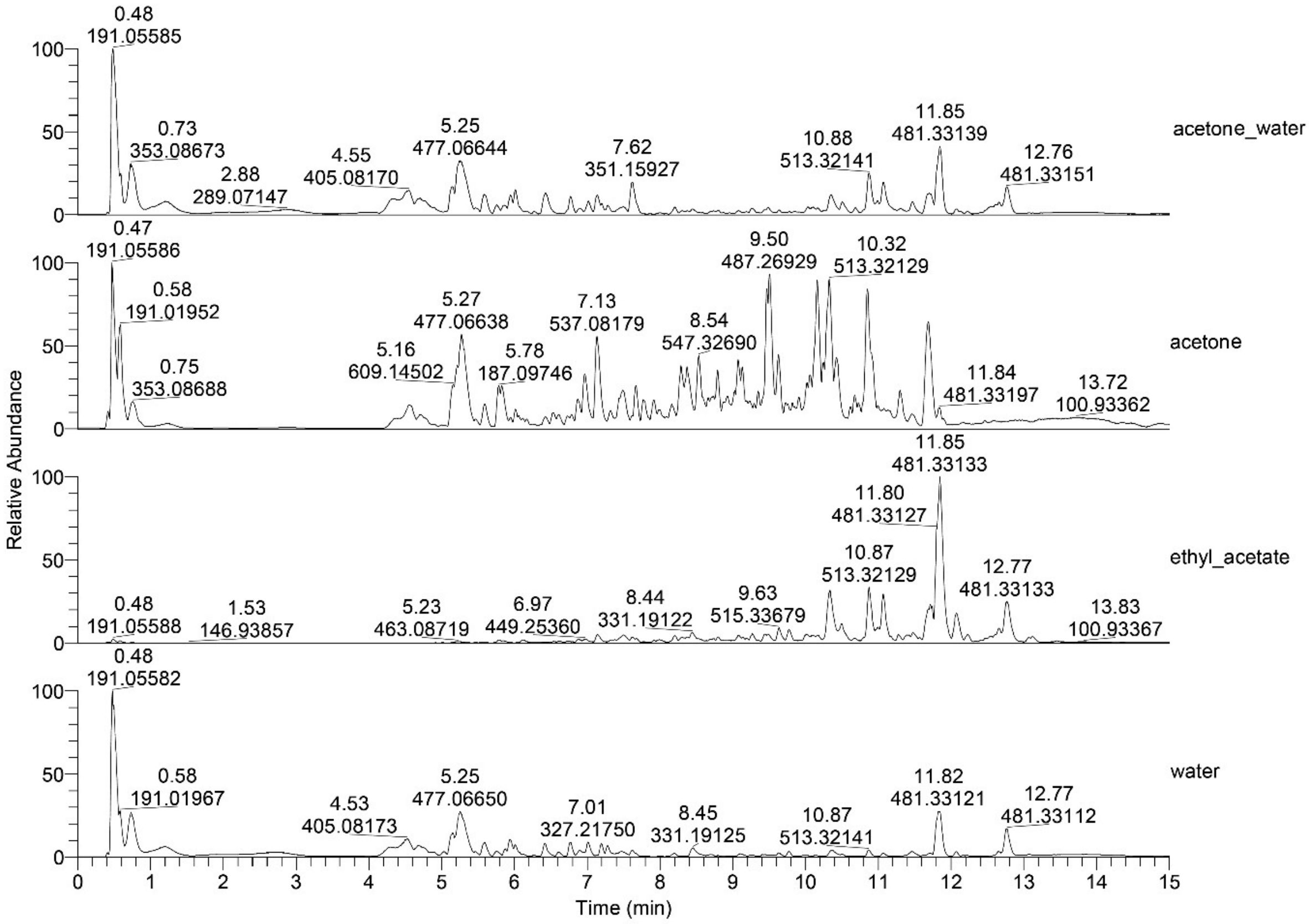

Figure S2. Base peak chromatograms of *Hypericum* samples obtained with different extraction solvents.

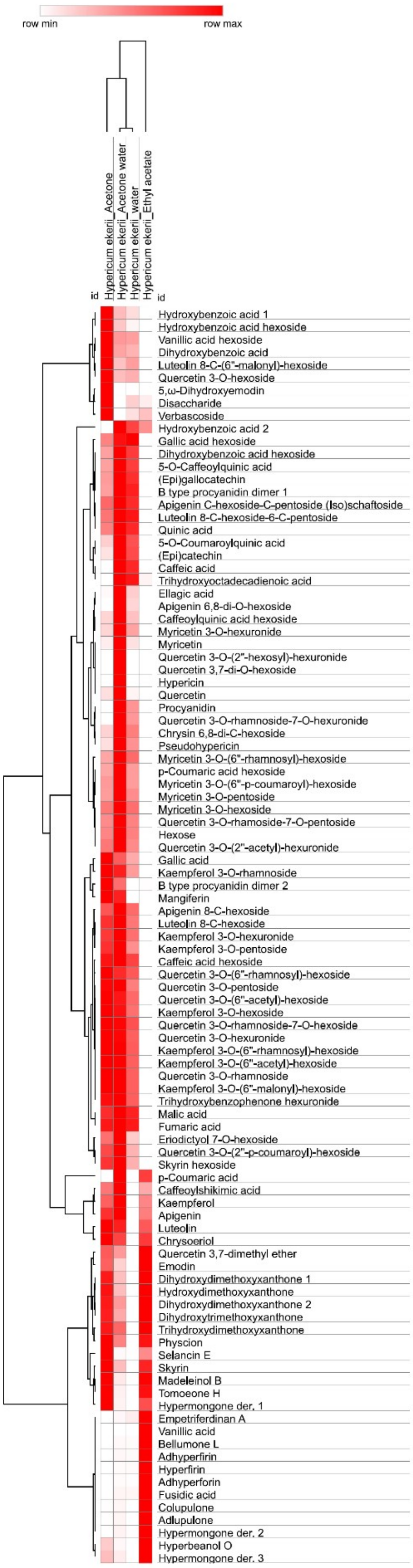

Figure S3. Heatmap of the scaled data of untargeted metabolite analysis, with the samples (both columns and rows) arranged according to the HCA (Pearson method of cluster agglomeration). Intensity of red colour indicates the abundance of the compounds in samples, as indicated in the colour scale.

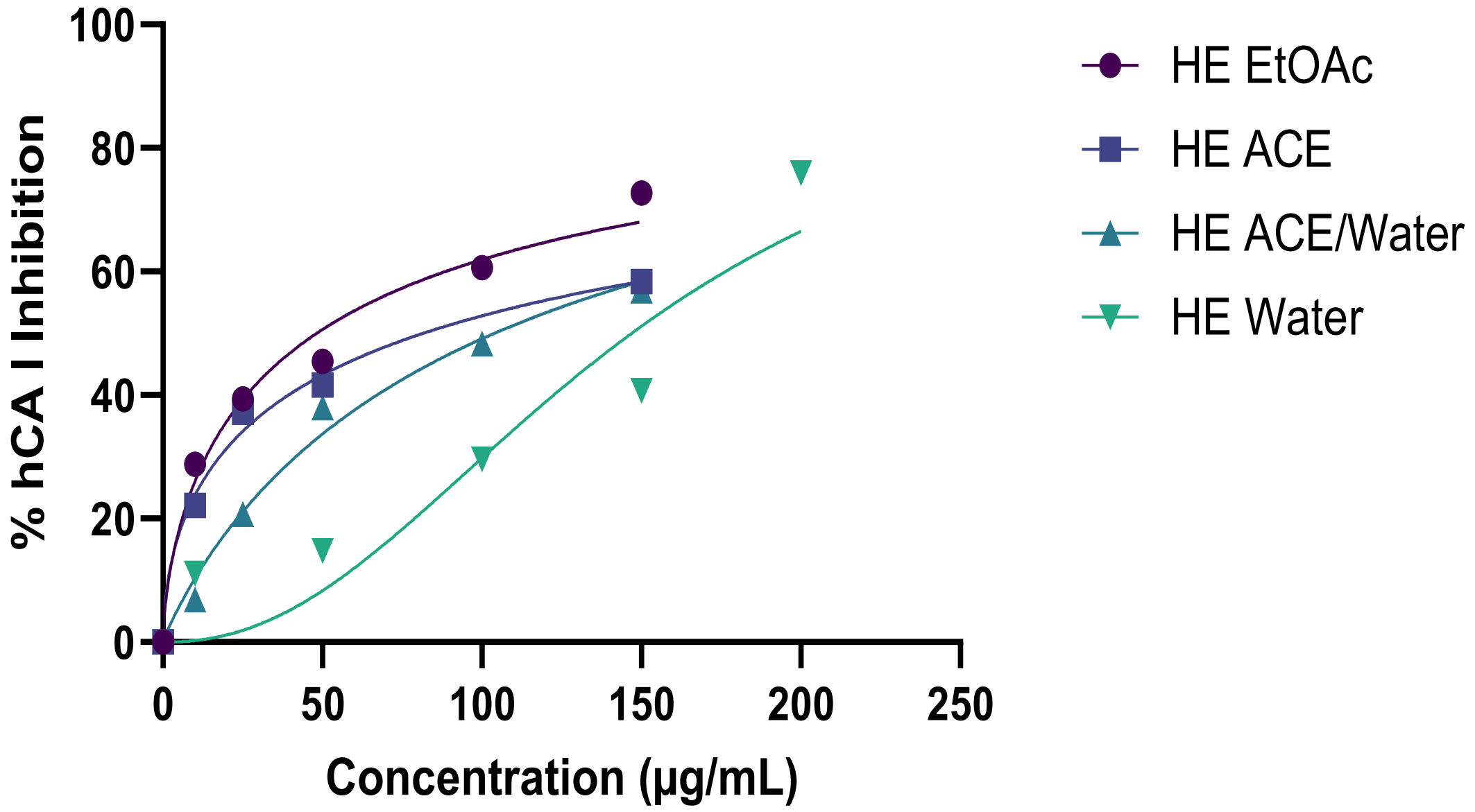

Figure S4. hCA I Inhibition graph of *H. ekerii* extracts; EtOAc, Ethylacetate, ACE, acetone. Results were expressed as µg/mL.

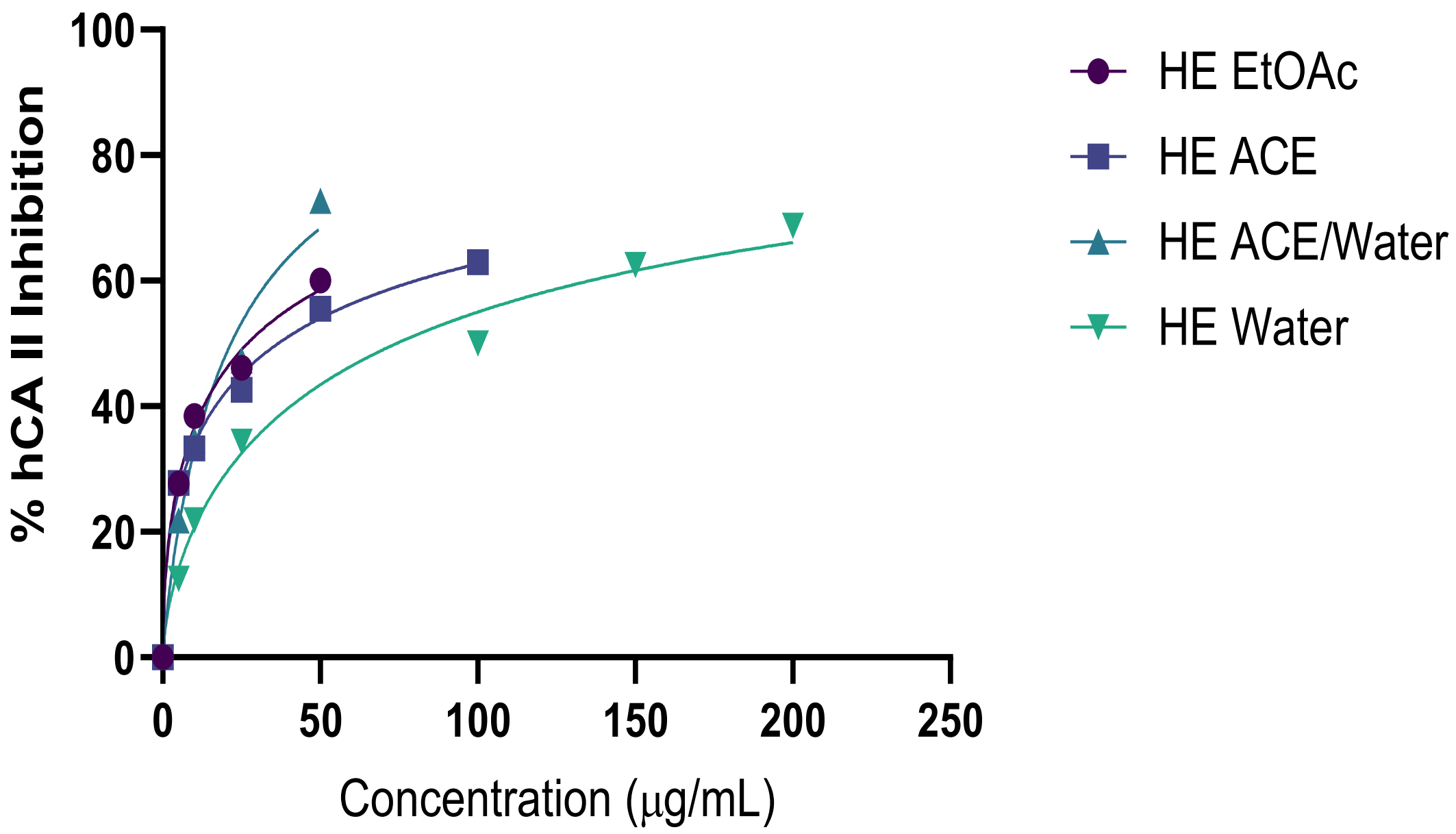

Figure S5. hCA II inhibition graph of *H. ekerii extracts;* EtOAc, Ethylacetate, ACE, acetone. Results were expressed as µg/mL.

References

1. A. J. Kirby and R. J. Schmidt, Journal of ethnopharmacology, 1997, 56, 103–108.
2. R. Re, N. Pellegrini, A. Proteggente, A. Pannala, M. Yang and C. Rice-Evans, Free radical biology and medicine, 1999, 26, 1231–1237.
3. R. Apak, K. Güçlü, M. Özyürek and S. E. Karademir, Journal of agricultural and food chemistry, 2004, 52, 7970–7981.
4. I. F. Benzie and J. J. Strain, Analytical biochemistry, 1996, 239, 70–76.
5. P. Prieto, M. Pineda and M. Aguilar, Analytical biochemistry, 1999, 269, 337–341.
6. T. C. Dinis, V. M. Madeira and L. M. Almeida, Archives of biochemistry and biophysics, 1994, 315, 161–169.
7. G. L. Ellman, K. D. Courtney, V. Andres Jr and R. M. Featherstone, Biochemical pharmacology, 1961, 7, 88–95.
8. T. Masuda, D. Yamashita, Y. Takeda and S. Yonemori, Bioscience, biotechnology, and biochemistry, 2005, 69, 197–201.
9. I. Šafašík, Journal of Enzyme Inhibition, 1990, 3, 245–247.
10. L. Ting, X.-d. Zhang, Y.-w. Song and J.-w. Liu, Chinese Journal of Clinical Pharmacology and Therapeutics, 2005, 10, 1128.
11. H. Karagecili, M. A. Yılmaz, A. Ertürk, H. Kiziltas, L. Güven, S. H. Alwasel and İ. Gulcin, Molecules, 2023, 28, 1739.
12. S. Demirci Kayıran, Ö. F. Tavlı, E. Mataracı Kara, A. Kaplan, H. Şahin, M. Boğa and E. Eroğlu Özkan, Frontiers in Pharmacology, 2025, 16, 1618761.
13. J. Qian, W. Nie, Z. Zhang, G. Fang, C. Li and W. Li, New Journal of Chemistry, 2025, 49, 15709–15722.
14. H. Rao, S. Liu, H. Wu, W. Wang, W. Wang, W. Su and P. Li, Viruses, 2025, 17, 900.
15. C. H. C. dos Santos, P. Stark, P. Rizzo, K. Franke and L. Wessjohann, Phytochemistry Letters, 2023, 57, 11–15.
16. S. Dall'Acqua, G. Ak, K. I. Sinan, F. Elbasan, I. Ferrarese, S. Sut, E. Yıldıztugay, G. Peron, E. Schievano and M. C. Nancy Picot-Allain, Frontiers in pharmacology, 2021, 12, 660735.
17. D. Kruszka, R. K. Selvakesavan, P. Kachlicki and G. Franklin, Industrial Crops and Products, 2022, 178, 114561.
18. M. M. Sherif, H. H. Elshikh, M. M. Abdel-Aziz, M. M. Elaasser and M. Yosri, BioMed research international, 2023, 2023, 6934398.
19. Z. Zhang, Z. Ying, M. He, Y. Zhang, W. Nie, Z. Tang, W. Liu, J. Chen, J. Ye and W. Li, Journal of Pharmaceutical and Biomedical Analysis, 2024, 248, 116313.
20. S. Srivastava, S. Srivastava, M. Rehman, R. Chaudhary, V. Agarwal, A. S. Kausik and V. Mishra, 2025.
21. H. Javrushyan, M. Ginovyan, T. Harutyunyan, S. Gevorgyan, Z. Karabekian, A. Maloyan and N. Avtandilyan, PloS one, 2025, 20, e0303736.
22. N. Tocci, D. Perenzoni, D. Iamonico, F. Fava, T. Weil and F. Mattivi, Frontiers in pharmacology, 2018, 9, 382.
23. O. Bender, E. J. Llorent-Martínez, G. Zengin, A. Mollica, R. Ceylan, L. Molina-García, M. L. Fernández-de Córdova and A. Atalay, PloS one, 2018, 13, e0197815.
24. J. G. Diaz, Journal of Molecular Structure, 2022, 1248, 131447.

25. S. Zhang, Y. Wang, Z. Cui, Q. Li, L. Kong and J. Luo, Plant Physiology and Biochemistry, 2023, 197, 107643.
26. I. F. Pérez-Ramírez, M. A. Gallegos-Corona, M. L. González-Dávalos, O. Mora, N. E. Rocha-Guzmán and R. Reynoso-Camacho, European Journal of Lipid Science and Technology, 2019, 121, 1800403.
27. T. Marinov, Z. Kokanova-Nedialkova and P. Nedialkov, Pharmacia, 2024, 71, 1–11.
28. D.-X. Jin, J.-F. He, K.-Q. Zhang and N.-Y. Zhang, Natural Product Research, 2024, 38, 781–788.
29. Z. Zhang, W. Nie, Y. Zhang, M. He, C. Li, S. Zhang and W. Li, Phytochemical Analysis, 2025, 36, 793–804.
30. S. Yigitkan, M. Akdeniz, A. Ertas, I. Yener, H. Alkan, M. A. Yilmaz, F. Aydin, S. O. Kocakaya, P. Y. Koseoglu and U. Kolak, Chemistry & Biodiversity, 2026, 23, e01956.
31. K. Kimáková, A. Kimáková, J. Idkowiak, M. Stobiecki, P. Rodziewicz, Ł. Marczak and E. Čellárová, Analytical and bioanalytical chemistry, 2018, 410, 7689–7699.
32. M. Pradeep, M. Saxena, D. Mondal and G. Franklin, Chemosphere, 2024, 356, 141789.
33. F. Chimenti, F. Cottiglia, L. Bonsignore, L. Casu, M. Casu, C. Floris, D. Secci, A. Bolasco, P. Chimenti and A. Granese, Journal of natural products, 2006, 69, 945–949.
34. X.-M. Li, X.-G. Luo, C.-L. Si, N. Wang, H. Zhou, J.-F. He and T.-C. Zhang, European Journal of Medicinal Chemistry, 2015, 96, 436–444.
35. A. Alahmad, I. Alghoraibi, R. Zein, S. Kraft, G. Drager, J.-G. Walter and T. Scheper, ACS omega, 2022, 7, 13475–13493.
36. A. Bernardi, C. López-alarcón, A. Aspee, S. Rech, G. Von Poser, R. Bride and E. Lissp, Journal of the Chilean Chemical Society, 2007, 52, 1326–1329.
37. D. Qiu, M. Zhou, T. Lin, J. Chen, G. Wang, Y. Huang, X. Jiang, W. Tian and H. Chen, Journal of natural products, 2019, 82, 1072–1080.
38. W. Ma, F. Ren, X. Yan, X. Wang, T. Wu and N. Li, Fitoterapia, 2024, 172, 105745.
39. N. Tanaka, Y. Kashiwada, S.-Y. Kim, M. Sekiya, Y. Ikeshiro and Y. Takaishi, Phytochemistry, 2009, 70, 1456–1461.
40. M.-I. Chung, J.-R. Weng, J.-P. Wang, C.-M. Teng and C.-N. Lin, Planta medica, 2002, 68, 25–29.
41. P. T. Nedialkov, D. Zheleva-Dimitrova, U. Girreser and G. M. Kitanov, Natural Product Research, 2007, 21, 1056–1060.
42. S. A. Fobofou, K. Franke, A. Porzel, W. Brandt and L. A. Wessjohann, Journal of Natural Products, 2016, 79, 743–753.
43. A. Kotland, J. Thiery and J. Hubert, Phytochemical Analysis, 2024, 35, 391–400.
44. W.-J. Xu, M.-D. Zhu, X.-B. Wang, M.-H. Yang, J. Luo and L.-Y. Kong, Journal of natural products, 2015, 78, 1093–1100.
45. Y.-R. Li, W.-J. Xu, S.-S. Wei, W.-J. Lu, J. Luo and L.-Y. Kong, Phytochemistry, 2019, 159, 56–64.
46. M. Gaid, J. Füller and C. Müller-Goymann, Planta Medica, 2019, 85, 591–598.
47. X. Zhou, W. Xu, Y. Li, M. Zhang, P. Tang, W. Lu, Q. Li, H. Zhang, J. Luo and L. Kong, Journal of agricultural and food chemistry, 2021, 69, 646–654.

48. N. Y. Bektaş, E. Ersoy, M. Boğa, T. Boran, E. Cinar, G. Özhan, A. C. Gören and E. E. Özkan, Turkish journal of chemistry, 2021, 45, 1621–1638.
49. E. L. Schymanski, J. Jeon, R. Gulde, K. Fenner, M. Ruff, H. P. Singer and J. Hollender, 2014.
